# The COVID-19 pandemic’s true death toll in Iran after two years: an interrupted time series analysis of weekly all-cause mortality data

**DOI:** 10.1186/s12889-023-15336-0

**Published:** 2023-03-07

**Authors:** Reza Ebrahimoghli, Abbas Abbasi-Ghahramanloo, Eslam Moradi-Asl, Davoud Adham

**Affiliations:** Department of Public Health, School of Health, Ardabil University of Medical Sciences, Ardabil, Iran

**Keywords:** COVID-19, Mortality, Interrupted Time Series Analysis, Iran

## Abstract

**Introduction:**

: This study aimed to investigate overall and age group/region/sex-specific excess all-cause mortality from the inception of the COVID-19 pandemic in Iran until February 2022.

**Methods:**

Weekly all-cause mortality data were obtained for the period March 2015 until February 2022. We conducted interrupted time series analyses, using a generalized least-square regression model to estimate excess mortality after the COVID-19 pandemic. Using this approach, we estimated the expected post-pandemic death counts based on five years of pre-pandemic data and compared the results with observed mortality during the pandemic.

**Results:**

After the COVID-19 pandemic, we observed an immediate increase (1,934 deaths per week, p = 0.01) in weekly all-cause mortality. An estimated 240,390 excess deaths were observed in two years after the pandemic. Within the same period, 136,166 deaths were officially attributed to COVID-19. The excess mortality was greatest among males compared with females (326 versus 264 per 100k), with an increasing trend by age group. There is a clear increased excess mortality in the central and northwestern provinces.

**Conclusion:**

We found that the full mortality burden during the outbreak has been much heavier than what is officially reported, with clear differences by sex, age group, and geographical region.

**Supplementary Information:**

The online version contains supplementary material available at 10.1186/s12889-023-15336-0.

## Introduction

Iran has been one of the countries worst hit by the coronavirus disease 2019 (COVID-19) pandemic [1, 2]. The country had 6.7 million cases of COVID-19, the twelfth most in the world, with 133 thousand officially confirmed deaths from the disease as of Feb 11, 2022. As the pandemic progresses, precise measurement of its burden, across different population groups and geographical regions, should be a priority [3]. Estimating the mortality burden of the pandemic is pivotal to inform resource allocation and evaluation of public health interventions [4]. For example, an accurate understanding of the mortality burden of the pandemic is essential in considering the trade-offs that underlie political decision making (such as public health interventions or economic interventions) that must be integrated into pandemic responses at the national level [5]. This is particularly true in countries heavily affected by the pandemic such as Iran.

COVID-19 pandemic may cause excess mortality both in those infected (direct effects) and those affected (indirectly, not infected) by disruption of health services; the psychological, physical, and social consequences of distancing; and economic changes. Therefore, the mortality burden of COVID-19 cannot be completely captured by the analysis of officially reported COVID-19 deaths, which itself has also significant drawbacks as it is based on a limited testing capacity. One way to estimate and monitor the mortality burden of the COVID-19 pandemic is to estimate the difference between the observed number of all-cause deaths with those expected based on the background (pre-epidemic) morality pattern in the population [6]. This approach is considered a more reliable indicator of the mortality burden of the COVID-19 pandemic since it is less sensitive to testing capacity, coding errors, competing risks, and the potential misclassifications in designating the cause of deaths, and as such enables international comparisons [7, 8].

Recently, national authorities of countries have been urged to publicly release weekly all-cause mortality data at a high spatial and temporal resolution to facilitate the evaluation of mortality burden of the pandemic [9, 10]. According to these international recommendations, the National Organization for Civil Registration of Iran (NOCR) has replaced the previous style of reporting of aggregated seasonal all-cause mortality data with detailed weekly reporting of all-cause mortality by gender, age group, and geographical regions. This has made an unprecedented opportunity to investigate the mortality burden of the COVID-19 pandemic within the country.

Up to date, to the best of our knowledge, only two studies have investigated mortality burden of the COVID-19 pandemic in the country using a previous version of aggregated trimester mortality data [11, 12], while there are no comprehensive studies that have looked at the impact of COVID-19 on all-cause mortality trend using detailed weekly mortality data in term of regions, gender, and different age groups. Because of the limited number of yearly data points in these studies (i.e., one data point for every season), the use of rigorous statistical models to capture changes in mortality trends was not practically possible [13, 14]. Thus, the present study investigates overall and age-group/province/sex-specific excess all-cause mortality from the inception of the COVID-19 pandemic until February 2022 in Iran to understand the mortality burden of the pandemic in Iran.

## Methods

We conducted a retrospective, quasi-experimental, observational study on the effects of COVID-19 pandemic on the all-cause mortality trend in Iran. We used an interrupted time-series design to examine changes in mortality patterns following the COVID-19 pandemic. Specifically, we investigated the association of the COVID-19 pandemic with immediate and gradual changes in trends of mortality in Iran.

### Data

We used data from the NOCR, which contains weekly information regarding the number of all-cause deaths in Iran between March 2015 and February 2022. The choice of this period was based on widely adopted practices from previously published relevant studies. This dataset constitutes at least 92% of all deaths in the country [15]. Death counts were aggregated initially without stratification (national data) and then across age-group, gender, and province strata. We used the latest version of publicly available national and provincial census data (2016) to report our findings per 100k population.

### Statistical analysis

Interrupted time series analysis, as one of the most robust quasi-experimental designs [16], was used to estimate the effect of the COVID-19 pandemic on the all-cause mortality trend. This analytical approach uses time-series data (i.e., data collected through repeated measurements over time) before and after an exposure (e.g., COVID-19 pandemic) to establish a causal association between the exposure and an outcome of interest (e.g., death counts) [17].

As the first step, we presented descriptive statistics including a visual inspection of series over time and summary statistics before and after the COVID-19 outbreak in the country. Although the intuitive graphical presentation of results can often reflect potential changes in level and/or trend of the outcome of the interest, this approach implements a two-stage regression analysis to assess change and control for other potential effects. Therefore, we fitted a linear model using generalized least squares to each segment (i.e., pre-and post-pandemic period). Our regression model specification included the following terms:

$$\begin{gathered} Y\_t = \beta \_0 + \beta \_1t + \beta \_2X\_t + \beta \_3X\_t({\text{t}} - {\text{t}}\_0) \hfill \\\,\,\,\,\,\,\,\,\,\,\,\,\,\, + e\_t \hfill \\ \end{gathered} $$

Here, Y_t is the number of deaths in week t; t represent the time elapsed since the start of the observation in weeks; t_0 is the first time point of the pandemic; X_t is an indicator for time t occurring before (X_t = 0) or after (X_t = 1) the pandemic in the country. In this model, ${\beta }_{0}$ estimates the baseline level of the outcome, number of all-cause mortality per week, at time zero; $\beta \_1$estimates the change in the number of all-cause mortality that occurs each week before the COVID-19 (i.e. the baseline trend); $\beta \_2$ estimates the level change in the weekly number of all-cause mortality immediately after the COVID-19, that is, from the end of the preceding segment; and $\beta \_3$ estimates the change in the trend in the weekly number of all-cause mortality after the COVID-19 pandemic, compared with the weekly trend before the COVID-19. The sum of $\beta \_1$ and $\beta \_3$ is the post-intervention slope. The error term e_t at time t indicates the random variability not explained by the model.

To detect autocorrelation, we visually inspect a plot of residuals against time and we observed randomly scattered residuals, without a pattern, indicating a lack of autocorrelation [18]. We also used The Durbin–Watson statistic, to test for serial autocorrelation of the error terms in the regression model [19]. Adjustment for autocorrelation involves estimating the autocorrelation parameter and including it in the segmented regression model if necessary. We obtained the order of autocorrelation based on examining both the autocorrelation and partial autocorrelation functions. We also adjusted for seasonality by fitting Fourier terms (pairs of sine and cosine functions) into our model. We used pairs of sine and cosine functions of time with the underlying period reflecting the full seasonal cycle. This approach is particularly suited to capturing very regular seasonal patterns (1). in this regard, we only selected those Fourier terms that were found to be statistically significant in the regression model.

Separate models were fitted to examine excess mortality by sex, age group, and province. For post-COVID-19 weeks, from each model, we produced an estimate of the expected number of deaths as follows: we modelled data from pre-COVID-19 period, we estimated the underlying secular trend and extrapolated this estimated trend to the post-COVID-19 period and created a counterfactual for what would have occurred in the absence of the pandemic. We made statistical comparisons between the counterfactual and observed mortality pattern to estimate the overall burden of the pandemic.

We used R (version 4.1.2, R Foundation for Statistical Computing) and Stata (version 16, Stata Corp, Collage Station, Texas, USA) for this analysis. The level of statistical significance was set at a *p*-value of less than 0.05.

## Results

**Fig. 1:**
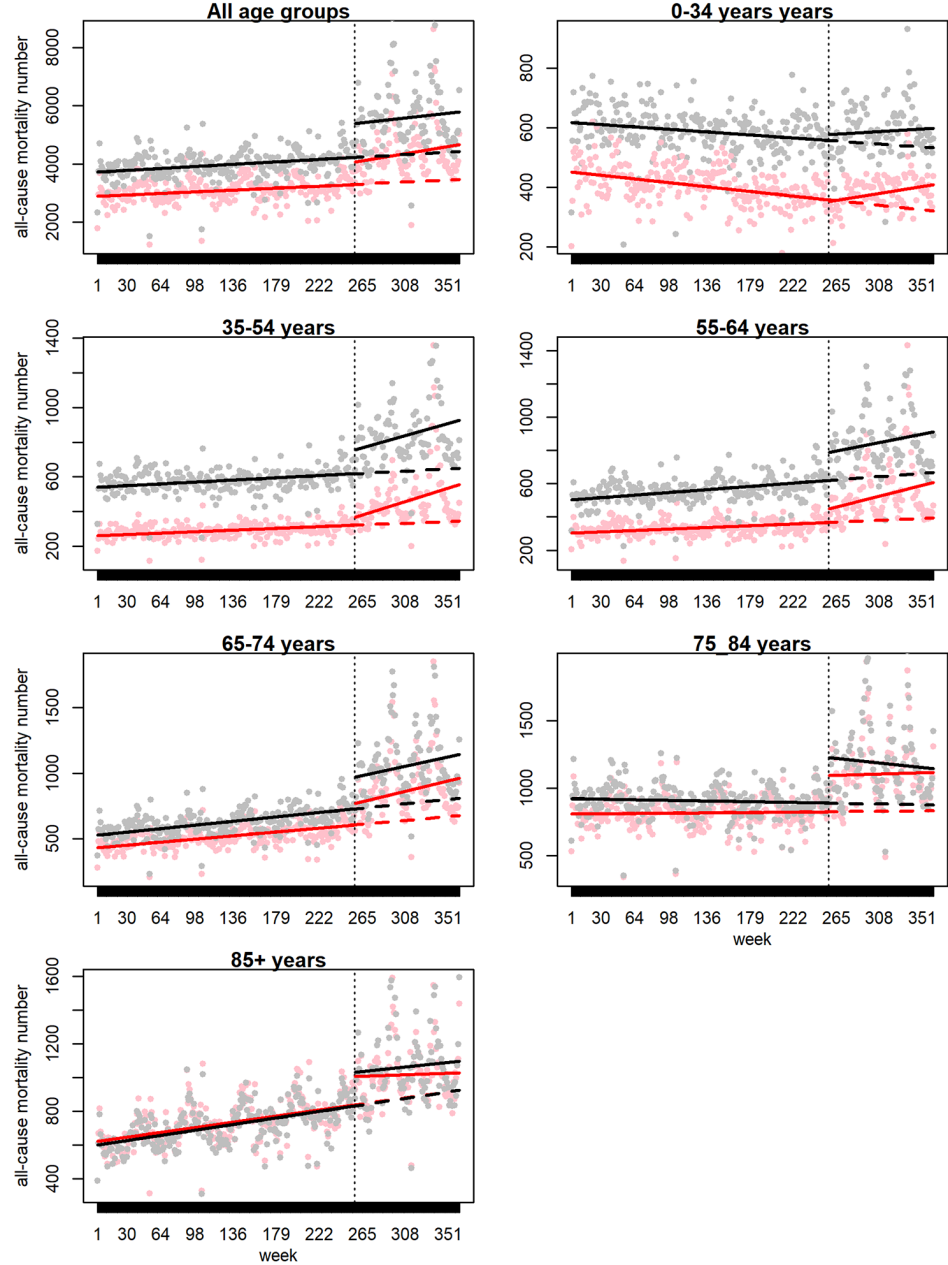
Observed (circles), predicted (solid lines), and counterfactual scenario (dash line) for male (black) and females (red)

The weekly all-cause mortality was higher during the COVID-19 pandemic compared with the average of the previous five years (10,001 versus 7,055). Before the COVID-19 pandemic, the total all-cause mortality had remained fairly constant for the past five years (p-value for the slope of the pre-COVID-19 trend line = 0.07). However, right after COVID-19, the estimated number of mortalities increased abruptly. The estimated average increase in the number of all-cause deaths was 2253 per week in the post-COVID-19 period (9,965 deaths) compared with expected death counts on trends in the five-year pre-COVID-19 (7,712) (p < 0.001). There was not a significant change in the week-to-week trend in the number of deaths after COVID-19 (p = 0·43). Figure 1 shows the scatterplot for the weekly mortality, with the superimposed regression-based time trends (See this figure with unsmoothed seasonal pattern in Supplementary File, Sect. 1).

By the last week of the observation period (February 19–25, 2022), we estimate that there were 240,390 more all-cause mortality than would have been expected if the pre-COVID-19 trend had continued (300 deaths per 100,000 population). Within the same period, 136,166 deaths were officially attributed to COVID-19 by the Ministry of Health. Table 1 presents data on estimated changes in level and trend of all-cause mortality as well as the number of excess deaths; overall and by age group and sex.

**Table 1:** Estimated effects of COVID-19 pandemic on all-cause deaths in Iran over two years

Age group	Sex	Pre-covid intercept	Pre-covid trend	Immediate level change	Post-covid trend	Expected post-covid mortality	Observed post-covid mortality	Total excess mortality	Excess mortality per 100k
All ages	**Both**	**6601**	**+ 3.583**	**1934** ^***^	**+ 6**	**809,767**	**1,050,157**	**240,390**	**300**
	Male	3716	+ 1.9	1164.5**	+ 1.8	454,774	588,988	134,214	331
	Female	2885	+ 1.5	773.7^***^	+ 4.1	354,869	461,169	106,300	269
0–34 yr	Male	618	− 0.2^***^	+ 19.0	+ 0.4^*^	57,329	61,755	4426	18
	Female	451	-0.3^***^	-6.3	+ 0.9^***^	35,632	40,055	4422	18
35–54 yr	Male	540	+ 0.3	+ 135.9^**^	+ 1.3	66,572	89,010	22,438	207
	Female	260	+ 0.2	+ 43.2	+ 1.5^*^	35,087	49,219	14,132	134
55–64 yr	Male	502	+ 0.4^*^	167.4^**^	+ 0.7	67,532	89,863	22,331	739
	Female	301	+ 0.2	+ 79.3	+ 1.2	39,891	56,471	16,580	517
65–74 yr	Male	529	+ 0.7^**^	+ 239^***^	+ 0.8	80,892	111,663	30,771	2164
	Female	434	+ 0.6^**^	+ 163.2^*^	+ 1.1	67,417	92,370	24,953	1555
75–84 yr	Male	920	-0.1	339.1^***^	-0.6	92,671	124,987	32,316	4003
	Female	809	+ 0.1	+ 269.7^***^	+ 0.1	86,953	116,418	29,465	3939
85 + yr	Male	597	+ 0.09^***^	+ 200.0^***^	-0.2	92,130	111,710	19,580	8474
	Female	619	+ 0.8^***^	+ 168.0^***^	-0.6	92,500	106,636	14,135	6501
^*^P < 0.05, ^**^P < 0.01, ^***^P < 0.001									

### Age and sex

**Fig. 2:**
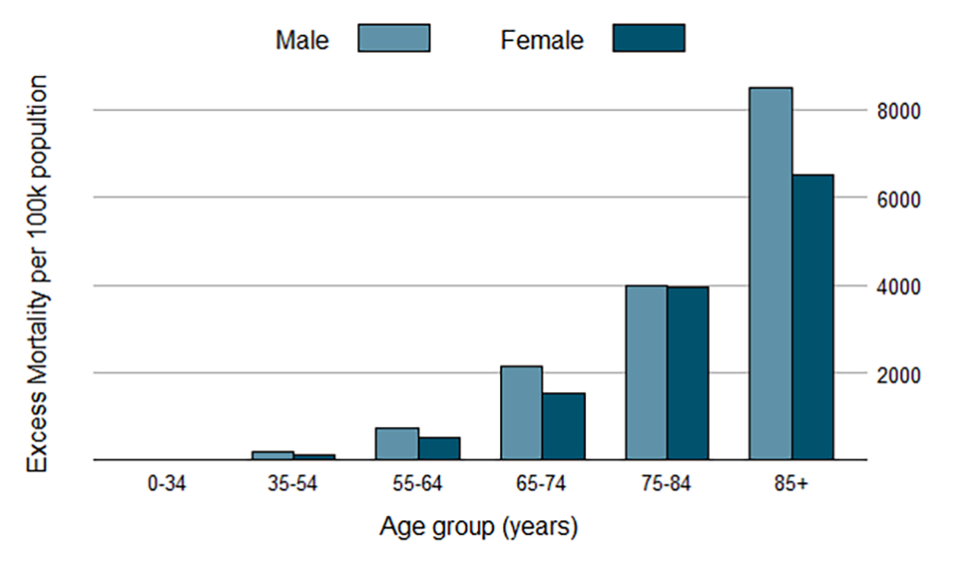
Age- and sex-specific excess deaths per 100 000 population in Iran from February 2020 to February 2022

We stratified the interrupted time-series analysis by sex. Both sexes had excess deaths. Overall excess mortality for men was 331 per 100k and for women was 269 per 100k. Excess all-cause deaths were also estimated for all males and females by age groups and were higher for men in almost all age groups. Figure 2 shows the number of excess deaths per 100k population by sex and age group.

We found excess deaths in all age groups. The statistically significant immediate effect was observed in all age groups, measured by whether the trend line shifted up at the time the COVID-19 pandemic was announced, but > 35 years old population (Table 1). However, for almost all age groups, the COVID-19 pandemic was not followed by a significant increase in the week-to-week mortality trend. The age groups with the largest number of excess deaths per 100k population were 85 years or over (with 8,447 per 100k in men and 6,501 per 100k in women) and 75–84 years (with 4,003 in men and 3,939 in women). In terms of the absolute number of excess mortalities, the population in the age group of 65–74 years had the greatest number of excess deaths at 55,724, followed by those with 75–84 years of age with 52,781 excess mortalities.

### Provinces

**Fig. 3:**
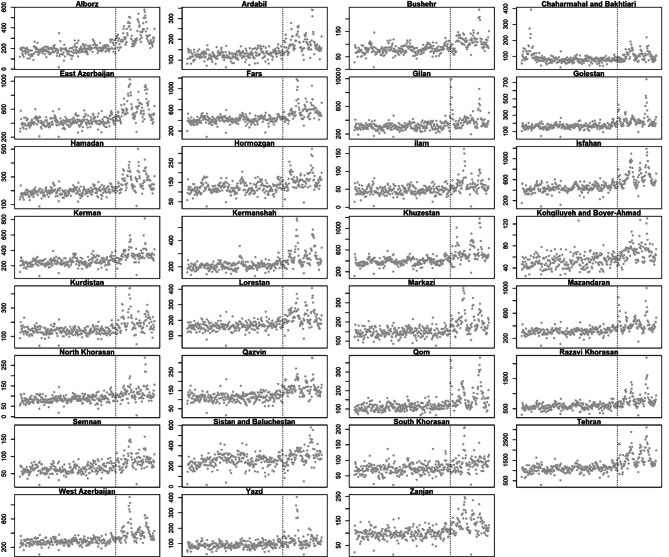
All-cause mortality trend before and after the COVID-19 pandemic in Iran, by province

**Fig. 4:**
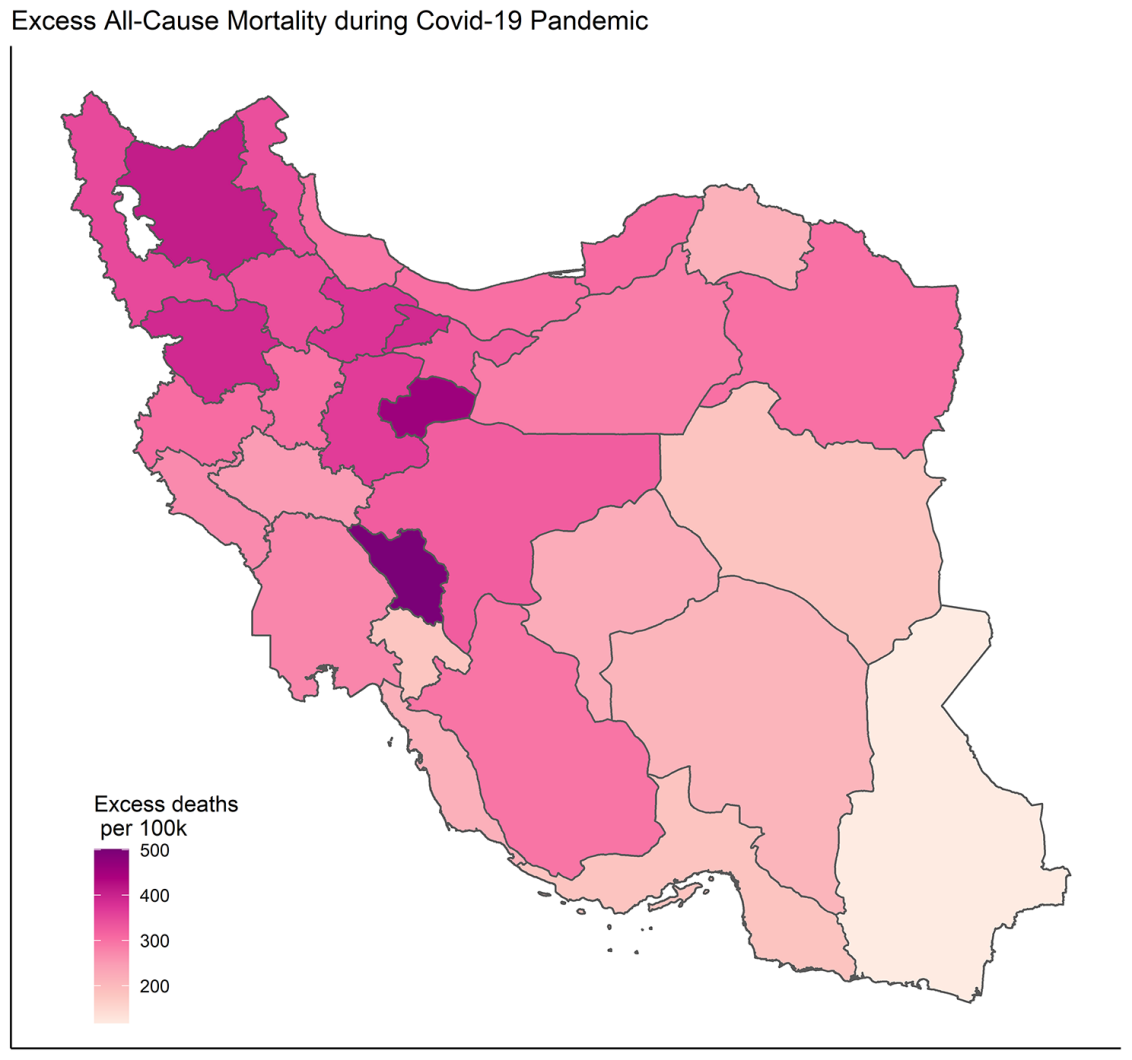
Geographic distribution of excess mortality after COVID-19 pandemic in Iran

All-cause mortality trends for provinces during our study periods are shown in Fig. 3. The extent of geographical differences is clear if looking at the excess mortality.

per 100k by province (Fig. 4). In the map, darker shades of colour indicate larger excess mortality per 100k after the pandemic. There is a clear increased darkening of the hot spots in the central and northwestern provinces.

Among the total population, the highest increase in all-cause mortality, in descending order, was observed for Chaharmahal and Bakhtiari, Qom, and East Azerbaijan (> 400 excess deaths per 100,000 population). In Sistan and Baluchestan, Kohgiluyeh and Boyer-Ahmad, and Hormozgan, the increase was less pronounced (< 184 excess deaths per 100,000 population; in ascending order).

## Discussion

Understanding the mortality burden of the COVID-19 pandemic is vital for public health decision making. The current study, comparing the pre-pandemic all-cause mortality trend with the post-pandemic trend showed that more than 240,000 Iranian people died due to the COVID-19 pandemic between the formal announcement of the outbreak in the country and February 2022, which is 1.8 times higher than the formally reported number of COVID-19 relate death.

The most obvious finding to emerge from our analyses is that the full mortality burden of the COVID-19 epidemic has been much heavier than what is reported by the official COVID-19 mortality surveillance. The official surveillance system reflects only a limited picture of the true death toll of the pandemic. A possible explanation for the observed gap between reported severe acute respiratory syndrome coronavirus 2 (SARSCoV-2) -related mortality and excess mortality may be due to underdiagnosis of the disease as a result of insufficient testing and reporting challenges. In line with this explanation, previous reports from Iranian health authorities suggest that a significant portion of suspected inpatient cases are not being tested for SARS-Cov-2 infection due to the limited number of test kits [20]; nevertheless, they are treated for SARS-Cov-2 infection based on their clinical symptoms. Another possible explanation for this is increased mortality from other diseases due to the pandemic-related disruptions in the provision of and access to routine health services.

The estimated excess all-cause mortality during the COVID-19 pandemic makes this disease one of the potential leading causes of death in Iran, considering pre-pandemic trends of other causes of death [21]. We were unable to divide the full mortality burden of the COVID-19 epidemic into those directly because of SARS-CoV-2 infection and deaths resulted from changes in other causes of death, due to the unavailability of national causes of deaths data with stratification by cause. Therefore, the results of this interrupted time series analysis quantify the full mortality burden of the COVID-19 pandemic in the country, not just the mortality burden directly due to SARS-CoV-2 infection.

We observed marked geographical differences in excess mortality burden in the country. The most interesting finding was that there is a strong northwest-to-southeast geographical gradient in the mortality burden of the COVID-19 pandemic in Iran. As it has been discussed in the literature, these regional differences may be partly due to seasonality patterns and more specifically the potential role of meteorological conditions influencing host susceptibility to infection and modes of transmission [22, 23]. There are, however, other possible explanations. The provinces with lower excess mortality are mainly provinces with lower population density. This finding accords with earlier observations, which showed that higher population density is significantly associated with both COVID-19 infection and mortality. Moreover, potential socio-economic variability at this geographical level may be meaningful [24, 25]. Also, variations in demographic and immunological profiles in the provinces, including age structure, may result in variations in mortality burden during this pandemic [26].

The current study found that the excess deaths were driven by excess deaths in both females and males. However, stratified analyses indicate differences in the overall mortality burden by sex. Mortality burden was higher in men compared with women across almost all age groups, consistent with previous COVID-19 mortality burden studies [27]. The reasons for such differences are still debated. This finding is likely to be related to the potential differences in exposure, susceptibility (e.g., as perhaps related to sex hormones), or health outcomes [28]. In addition, the debate over social aspects of gender (e.g., greater likelihood of healthy habits among women compared to men) and healthcare-seeking behaviours are long standing [29, 30]. In the case of COVID-19, robust evidence is needed to inform these debates.

The increased mortality burden with age observed in this analysis corroborates with numerous studies across the world investigating excess mortality due to the COVID-19 pandemic [27, 31]. Age is one of the main risk factors for COVID-19-related mortality. The progressive accumulation of multiple chronic conditions over ageing [32] increases individuals’ exposure to developing cognitive and physical impairments, increasing their vulnerability to the onset of infectious diseases and severe outcomes. But one unanticipated finding from this study is that more than a third of the absolute number of excess mortalities were in those younger than 65 years, indicating that excess mortality due to the COVID-19 pandemic is not just an issue for elder.

### Strengths and Limitations

To our knowledge, this is the first peer-reviewed report on excess mortality due to the COVID-19 pandemic in Iran using weekly all-cause mortality data. Our study benefits from the application of a rigorous and advanced interrupted time series analysis approach, allowing control of potential biases of time series data such as autocorrelation, seasonality, or non-stationarity which were not considered in most of the previous studies investigating the pandemic’s true death toll. In this analytical approach, we adjusted for baseline trends which helped us to control for most threats to internal validity. Sensitivity analysis suggests our results are robust. The results of this study were also robust to sensitivity analysis for assessing potential cofounding effect of changes in mean monthly temperature (supplementary file, Sects. 2–3) and type of regression models used (supplementary file, Sect. 4) on relationship between COVID-19 outbreak and excess all-cause mortality.

Some limitations must, however, be acknowledged. One important challenge in almost all excess mortality assessments is the issue of delays in reporting deaths within and between study countries, making it difficult to compare international findings. Therefore, national authorities should try to implement digital reporting of mortality data as well as rapid publishing of the data to the public health community. Implementation of such reporting systems not only facilitates tracking the outcomes of this pandemic and potential future outbreaks, but it makes it easier to investigate the effect of interventions on mortality burden. Another limitation of our results is that, due to the unavailability of cause-specific mortality data, we were unable to disentangle the specific causes of excess death during this pandemic.

## Conclusion

In conclusion, we have used an interrupted time series analysis approach to provide a detailed picture of excess all-cause mortality due to the COVID-19 pandemic during the first two-year period in Iran. The results of the present study suggest that the mortality burden of the ongoing COVID-19 pandemic has been more tremendous than reported by official SARS-CoV-2 infection. The official surveillance system reflects only a limited picture of the true death toll of the pandemic. We have pointed out that there are clear differences in excess mortality by sex, age group, and geographical region. Further work is required to establish the differential pattern across regions and subgroups and the potential impact of national or local interventions during the outbreak.

**Legends**.

## Electronic supplementary material

Below is the link to the electronic supplementary material.

Supplementary Material 1: The COVID-19 Pandemic?s True Death Toll in Iran after Two Years: An Interrupted Time Series Analysis of Weekly All-Cause Mortality Data

Supplementary Material 2: The COVID-19 Pandemic?s True Death Toll in Iran after Two Years: An Interrupted Time Series Analysis of Weekly All-Cause Mortality Data

## Data Availability

Anyone can visit and download needed data from the National Organization for Civil Registration of Iran web site: (https://www.sabteahval.ir).

## Abbreviations

COVID-19: Coronavirus Disease 2019
NOCR: National Organization for Civil Registration of Iran
SARSCoV-2: severe acute respiratory syndrome coronavirus 2

## References

[1] MohammadEbrahimi S, Mohammadi A, Bergquist R, Dolatkhah F, Olia M, Tavakolian A, Pishgar E, Kiani B (2021). Epidemiological characteristics and initial spatiotemporal visualisation of COVID-19 in a major city in the Middle East. BMC Public Health.

[2] Moradi-Asl E, Adham D, Ghobadi H, Abbasi-Ghahramanloo A (2021). Clustering of COVID-19 symptoms among iranian patients: the role of Preexisting Comorbidity on Latent Class Membership. Asia Pac J Public Health.

[3] United Nations Statistics Division. Demographic and social statistics. 2015. https://unstats.un.org/unsd/demographic-social/crvs/.

[4] Karlinsky A, Kobak D (2021). Tracking excess mortality across countries during the COVID-19 pandemic with the World Mortality dataset. Elife.

[5] Whittaker C, Walker PG, Alhaffar M, Hamlet A, Djaafara BA, Ghani A, Ferguson N, Dahab M, Checchi F, Watson OJ. Under-reporting of deaths limits our understanding of true burden of covid-19. *bmj* 2021, 375. 10.1136/bmj.n2239 34642172

[6] Banerjee A, Pasea L, Harris S, Gonzalez-Izquierdo A, Torralbo A, Shallcross L, Noursadeghi M, Pillay D, Sebire N, Holmes C (2020). Estimating excess 1-year mortality associated with the COVID-19 pandemic according to underlying conditions and age: a population-based cohort study. The Lancet.

[7] Islam N, Jdanov DA, Shkolnikov VM, Khunti K, Kawachi I, White M, Lewington S, Lacey B. Effects of covid-19 pandemic on life expectancy and premature mortality in 2020: time series analysis in 37 countries. *bmj* 2021, 375. 10.1136/bmj-2021-066768 PMC8564739 34732390

[8] Timonin S, Klimkin I, Shkolnikov VM, Andreev E, McKee M, Leon DA (2022). Excess mortality in Russia and its regions compared to high income countries: an analysis of monthly series of 2020. SSM-population health.

[9] Leon DA, Shkolnikov VM, Smeeth L, Magnus P, Pechholdová M, Jarvis CI (2020). COVID-19: a need for real-time monitoring of weekly excess deaths. The Lancet.

[10] Organization WH. Revealing the toll of COVID-19: a technical package for rapid mortality surveillance and epidemic response. 2020.

[11] Ghafari M, Kadivar A, Katzourakis A. Excess deaths associated with the Iranian COVID-19 epidemic: a province-level analysis. *medRxiv* 2020. 10.1016/j.ijid.2021.04.015 PMC8208896 33862214

[12] Tadbiri H, Moradi-Lakeh M, Naghavi M (2020). All-cause excess mortality and COVID-19-related deaths in Iran. Med J Islamic Repub Iran.

[13] Zhang J, Zhao Z, Xue Y, Chen Z, Ma X, Zhou Q. Time series analysis.Handbook of Medical Statistics2017,269.

[14] Mills TC. Applied time series analysis: a practical guide to modeling and forecasting. Academic press; 2019.

[15] Giaj Levra N, Filippi AR, Guarneri A, Badellino S, Mantovani C, Ruffini E, Ricardi U (2015). Efficacy and safety of stereotactic ablative radiotherapy in patients with previous pneumonectomy. Tumori.

[16] Jandoc R, Burden AM, Mamdani M, Lévesque LE, Cadarette SM (2015). Interrupted time series analysis in drug utilization research is increasing: systematic review and recommendations. J Clin Epidemiol.

[17] Cook TD, Campbell DT, Shadish W. Experimental and quasi-experimental designs for generalized causal inference. Houghton Mifflin Boston, MA; 2002.

[18] McLeod AI, Yu H, Mahdi E. Time series analysis with R. Handbook of statistics. Volume 30,edn.: Elsevier; 2012:pp. 661–712.

[19] Durban J, Watson G (1951). Testing for serial correlation in least squares regression. II Biometrika.

[20] Ghafari M, Kadivar A, Katzourakis A (2021). Excess deaths associated with the iranian COVID-19 epidemic: a province-level analysis. Int J Infect Dis.

[21] Murray CJ, Aravkin AY, Zheng P, Abbafati C, Abbas KM, Abbasi-Kangevari M, Abd-Allah F, Abdelalim A, Abdollahi M, Abdollahpour I (2020). Global burden of 87 risk factors in 204 countries and territories, 1990–2019: a systematic analysis for the global burden of Disease Study 2019. The Lancet.

[22] Audi A, AlIbrahim M, Kaddoura M, Hijazi G, Yassine HM, Zaraket H. Seasonality of respiratory viral infections: will COVID-19 follow suit?Frontiers in Public Health2020:576. 10.3389/fpubh.2020.567184 PMC7522168 33042956

[23] Achilleos S, Quattrocchi A, Gabel J, Heraclides A, Kolokotroni O, Constantinou C, Pagola Ugarte M, Nicolaou N, Rodriguez-Llanes JM, Bennett CM (2022). Excess all-cause mortality and COVID-19-related mortality: a temporal analysis in 22 countries, from January until August 2020. Int J Epidemiol.

[24] Bhadra A, Mukherjee A, Sarkar K (2021). Impact of population density on Covid-19 infected and mortality rate in India. Model Earth Syst Environ.

[25] Brandén M, Aradhya S, Kolk M, Härkönen J, Drefahl S, Malmberg B, Rostila M, Cederström A, Andersson G, Mussino E (2020). Residential context and COVID-19 mortality among adults aged 70 years and older in Stockholm: a population-based, observational study using individual-level data. The Lancet Healthy Longevity.

[26] O’Driscoll M, Ribeiro Dos Santos G, Wang L, Cummings DA, Azman AS, Paireau J, Fontanet A, Cauchemez S, Salje H (2021). Age-specific mortality and immunity patterns of SARS-CoV-2. Nature.

[27] Scortichini M, Schneider dos Santos R, De’Donato F, De Sario M, Michelozzi P, Davoli M, Masselot P, Sera F, Gasparrini A (2020). Excess mortality during the COVID-19 outbreak in Italy: a two-stage interrupted time-series analysis. Int J Epidemiol.

[28] Krieger N (2003). Genders, sexes, and health: what are the connections—and why does it matter?. Int J Epidemiol.

[29] Devlin H. Men are much more likely to die from coronavirus-but why.The Guardian2020,26.

[30] Noubani A, Diaconu K, Ghandour L, El Koussa M, Loffreda G, Saleh S (2020). A community–based system dynamics approach for understanding factors affecting mental health and health seeking behaviors in Beirut and Beqaa regions of Lebanon. Globalization and health.

[31] Stang A, Standl F, Kowall B, Brune B, Böttcher J, Brinkmann M, Dittmer U (2020). Jöckel K-H: excess mortality due to COVID-19 in Germany. J Infect.

[32] Ebrahimoghli R, Janati A, Sadeghi-Bazargani H, Hamishehkar H (2021). Chronic Diseases and Multimorbidity in Iran: a study protocol for the Use of Iranian Health Insurance Organization’s claims database to Understand Epidemiology, Health Service utilization, and patient costs. Health Serv Outcomes Res Method.

