# Supplementary material for: The COVID-19 pandemic’s true death toll in Iran after two years: an interrupted time series analysis of weekly all-cause mortality data
Source: BMC Public Health. 2023 Mar 7;23:442. doi: 10.1186/s12889-023-15336-0 (PMC9990579; doi:10.1186/s12889-023-15336-0)
Supplement: Supplementary file 1 — Supplementary Material 1: The COVID-19 Pandemic?s True Death Toll in Iran after Two Years: An Interrupted Time Series Analysis of Weekly All-Cause Mortality Data [file 12889_2023_15336_MOESM1_ESM.docx]

**Supplementary File**

**The COVID-19 Pandemic’s True Death Toll in Iran after Two Years: An Interrupted Time Series Analysis of Weekly All-Cause Mortality Data**

**Section 1: seasional pattern of all-caused mortality number during the observation period in Iran.**


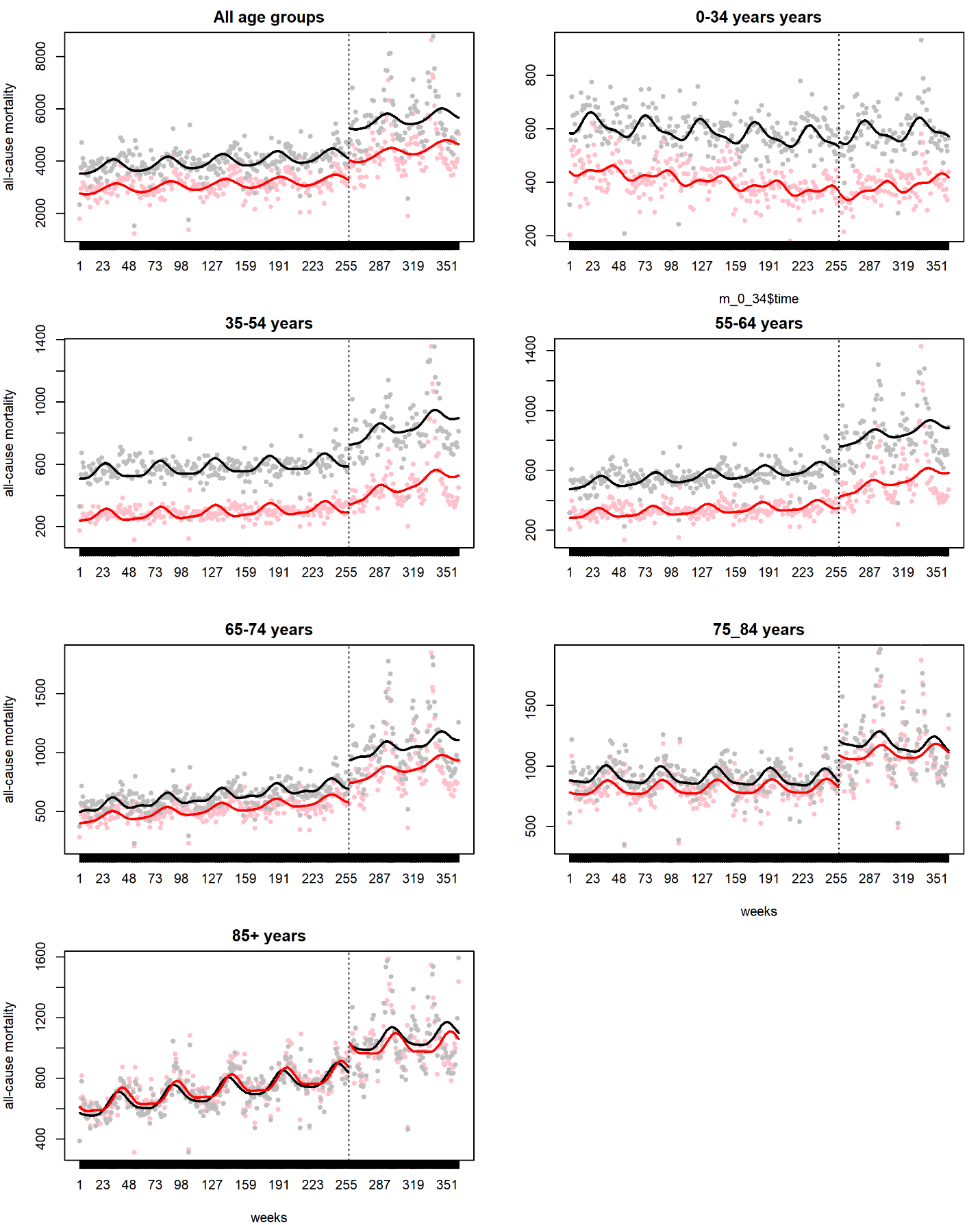


**Section 2: visual inspection of potential relationship between changes in monthly tempreture and monthly all-cause mortality**


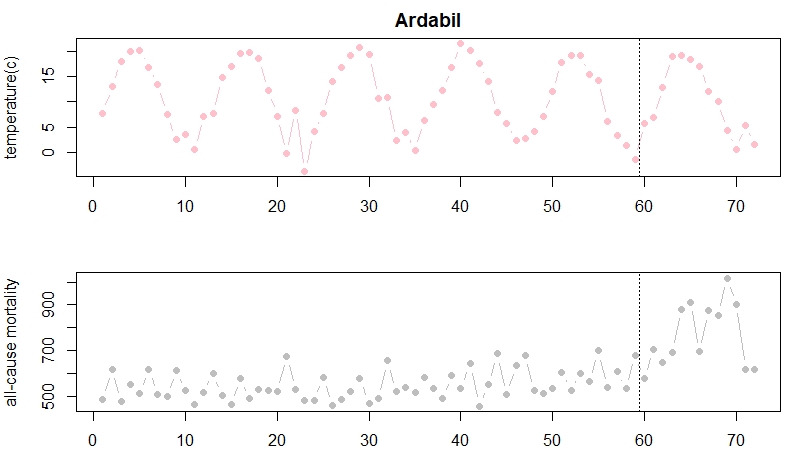


**months**


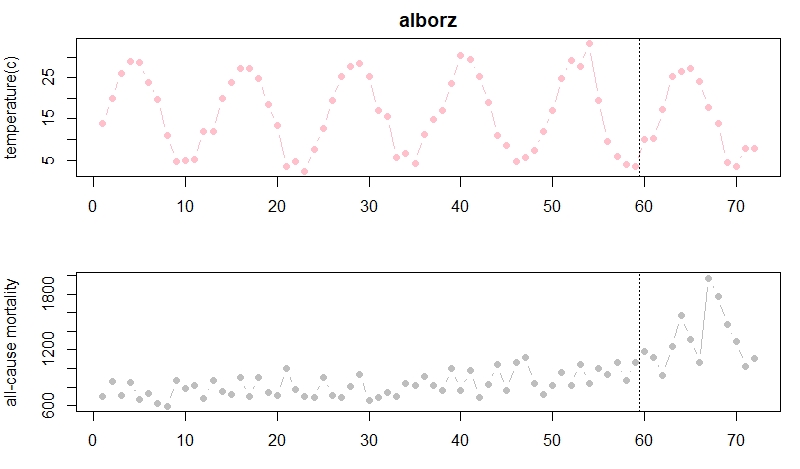


**months**


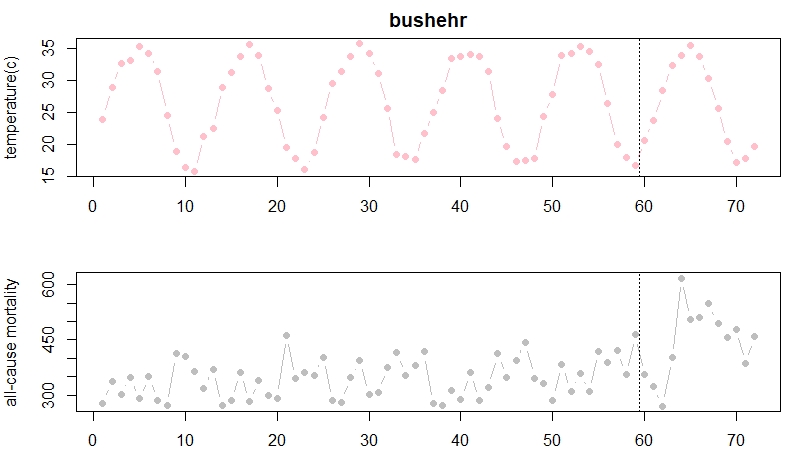


**months**


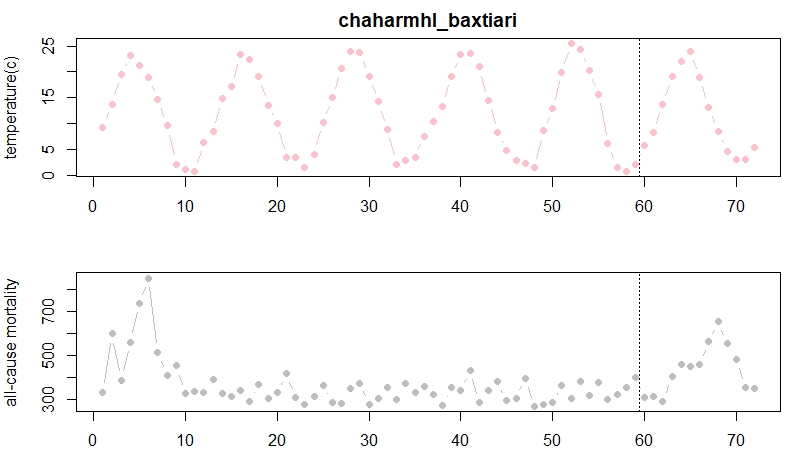


**months**


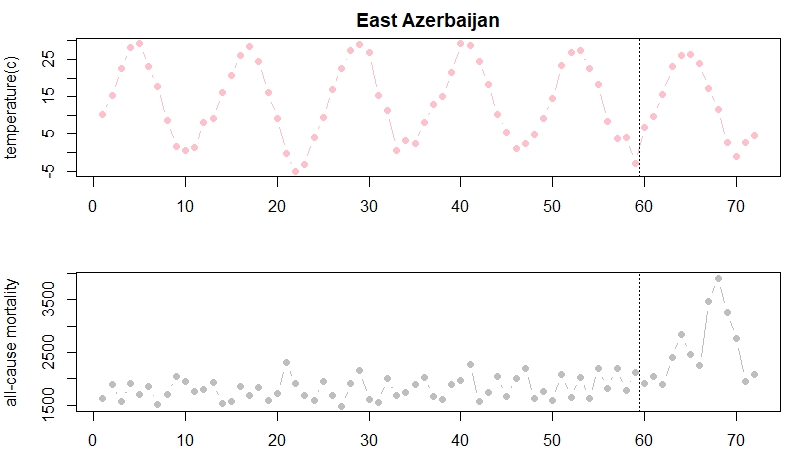


**months**


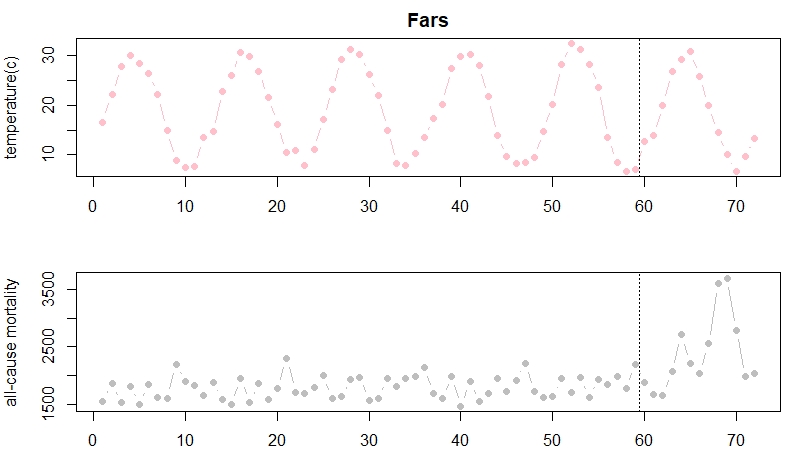


**months**


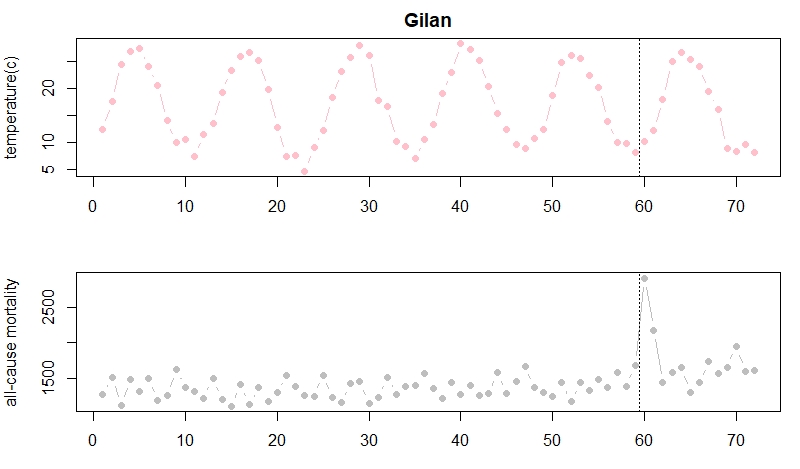


**months**


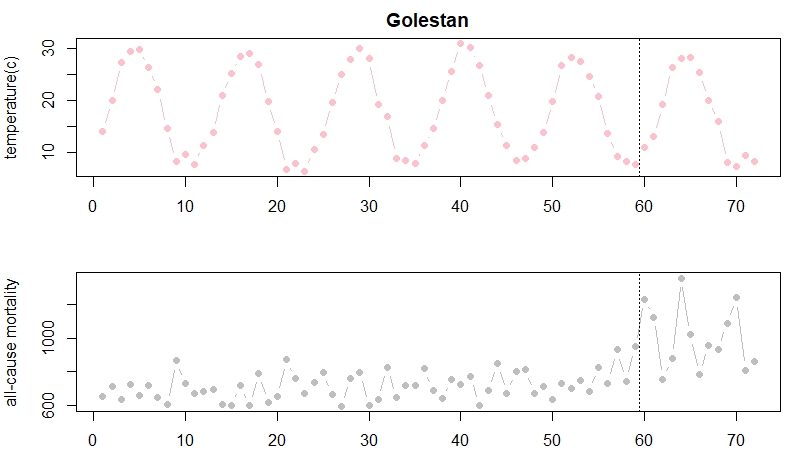


**months**


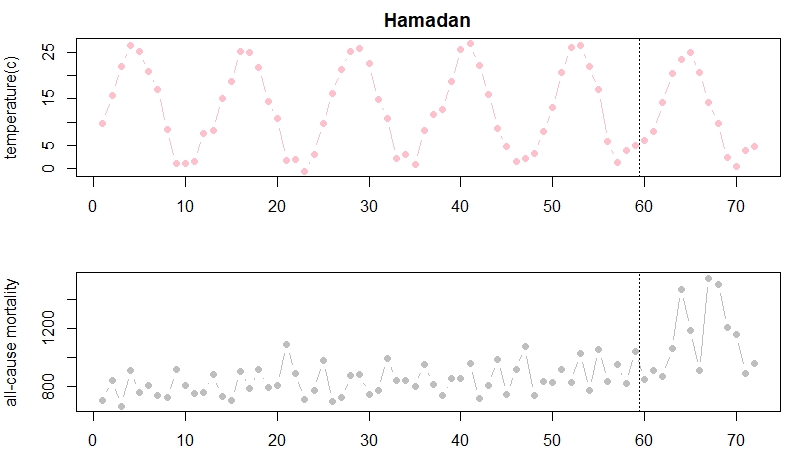


**months**


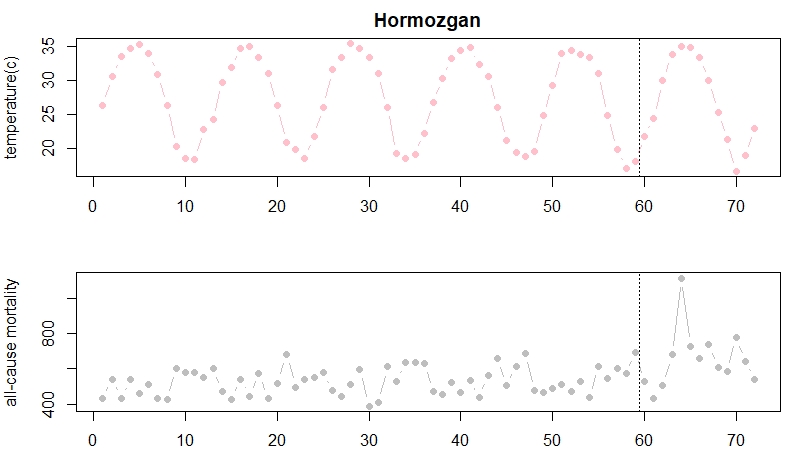


**months**


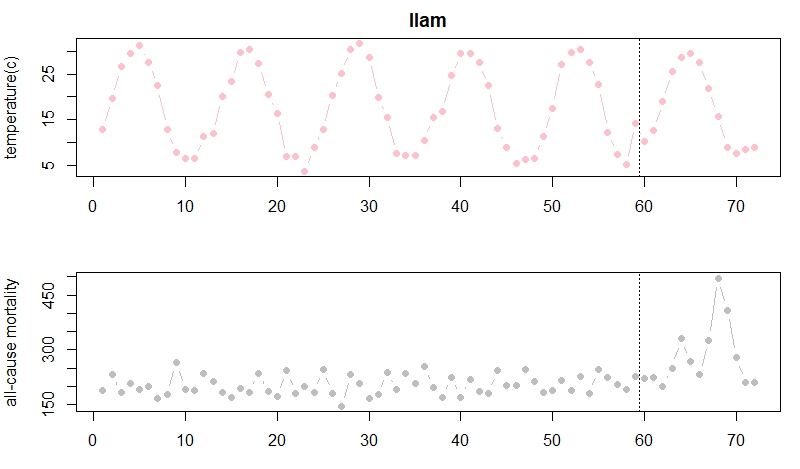


**months**


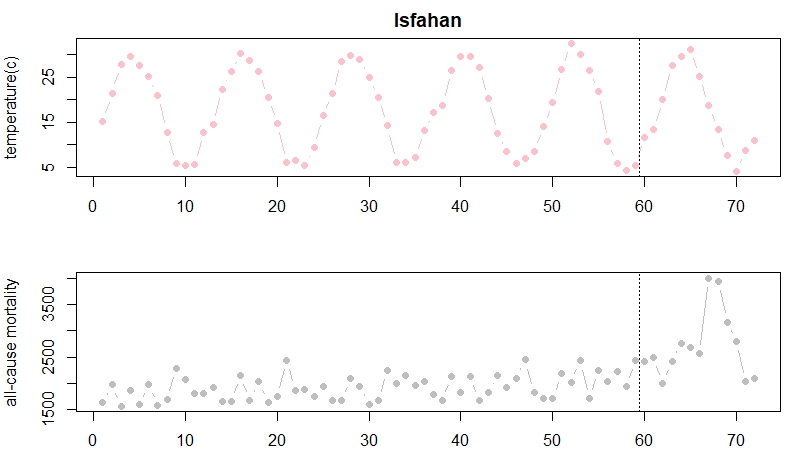


**months**


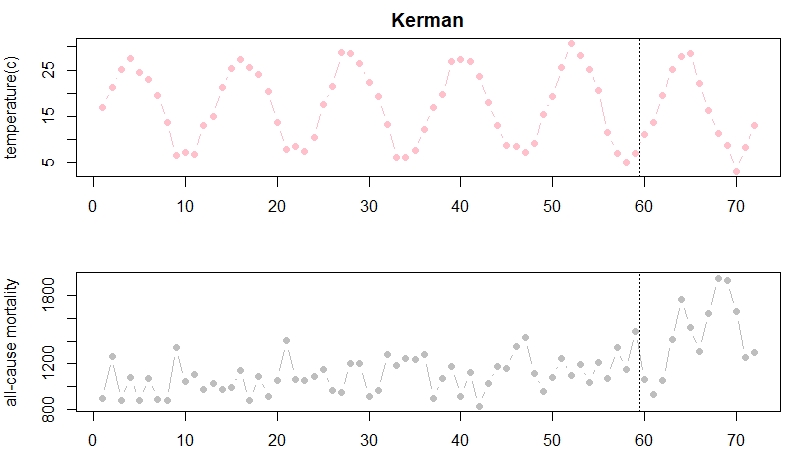


**months**


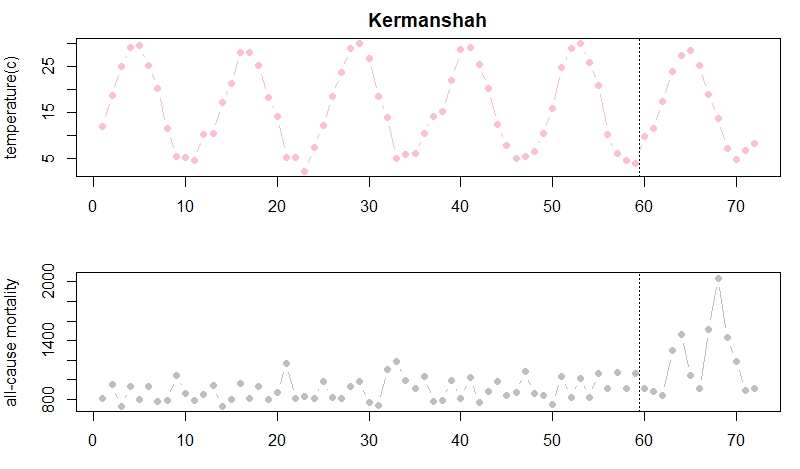


**months**


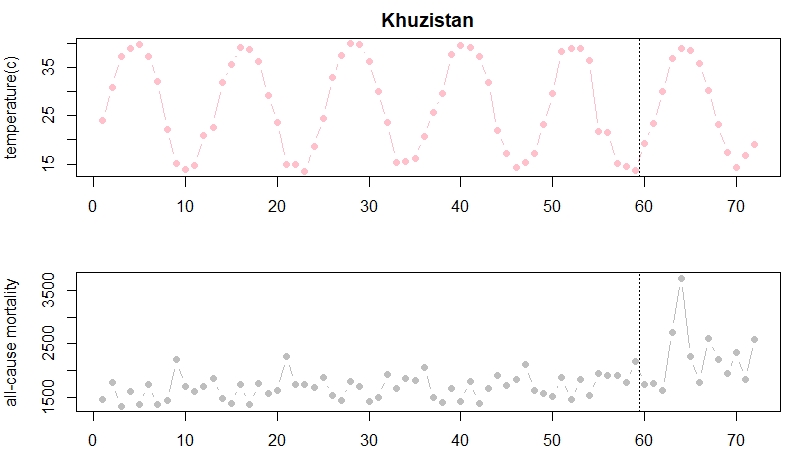


**months**


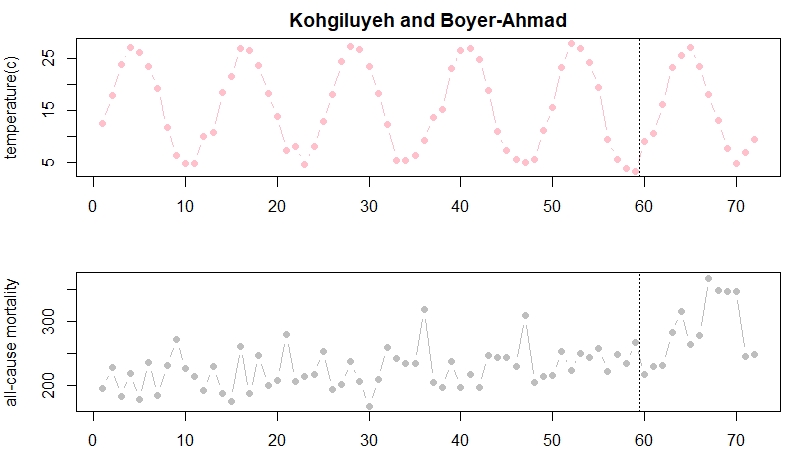


**months**


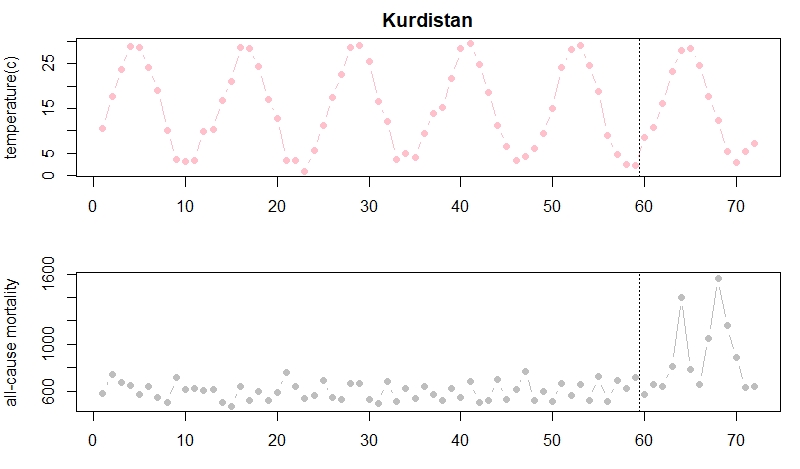


**months**


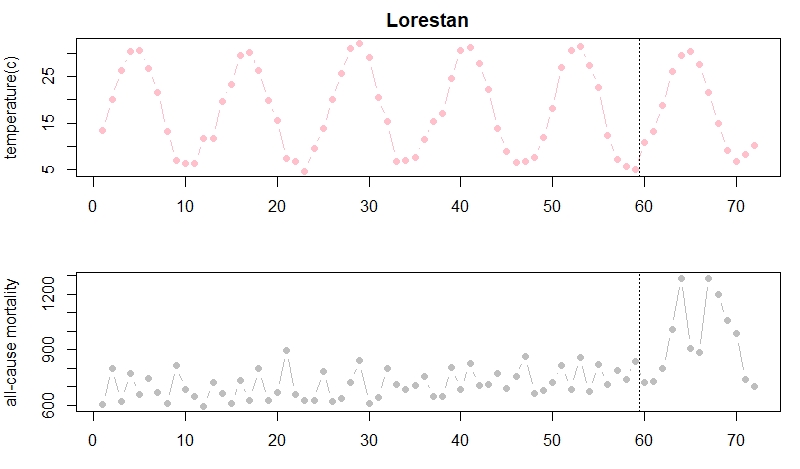


**months**


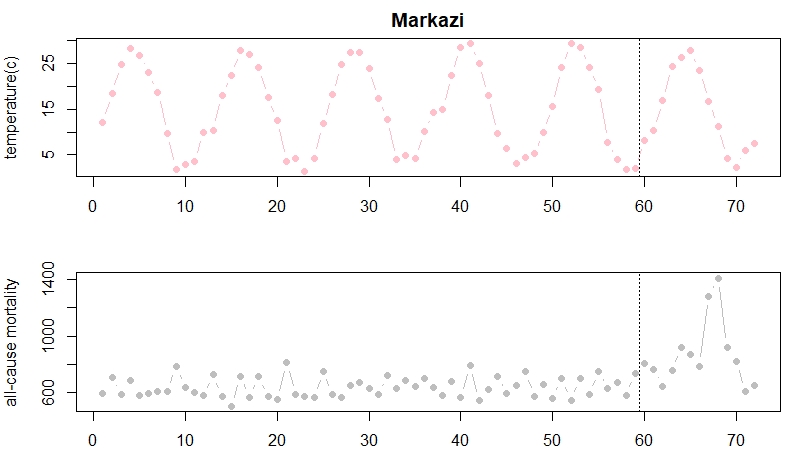


**months**


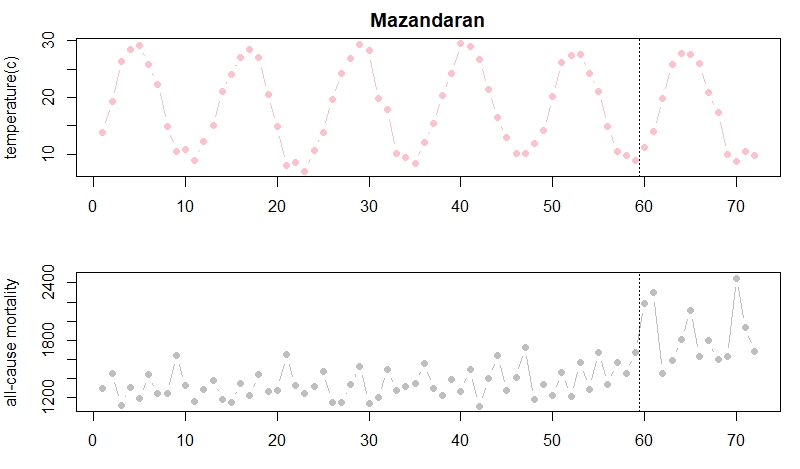


**months**


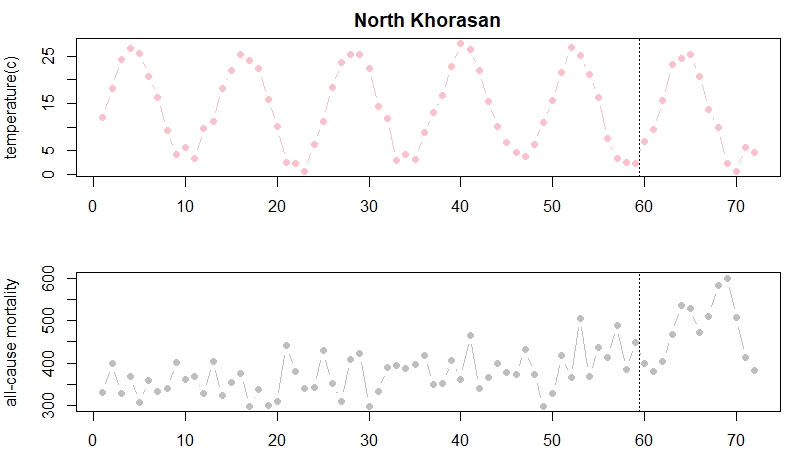


**months**


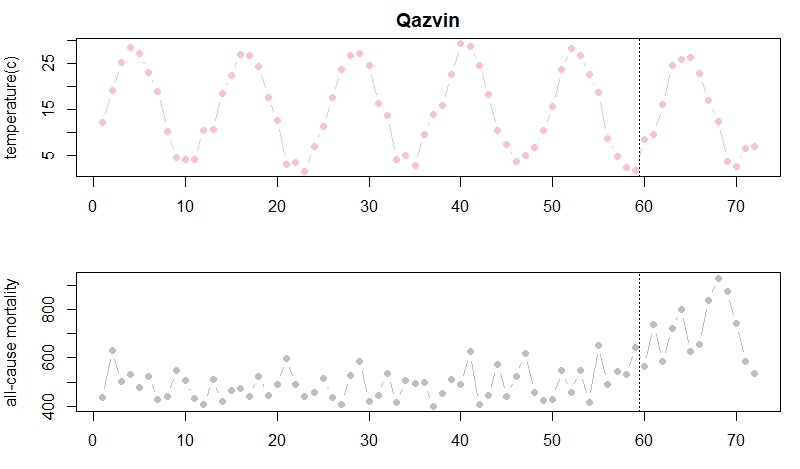


**months**


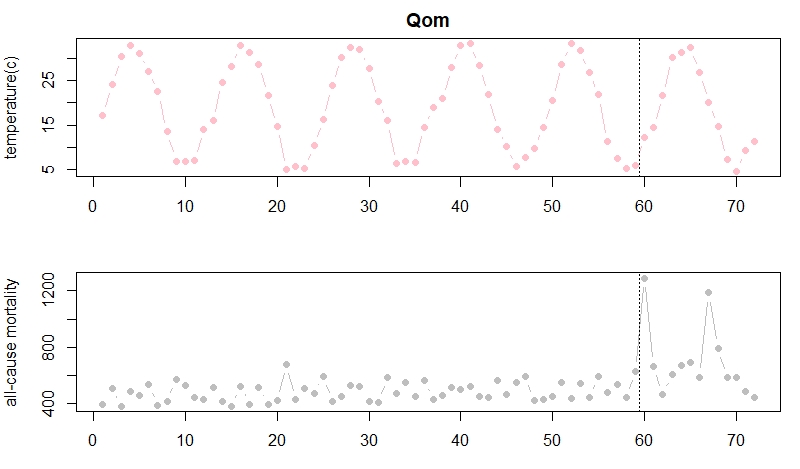


**months**


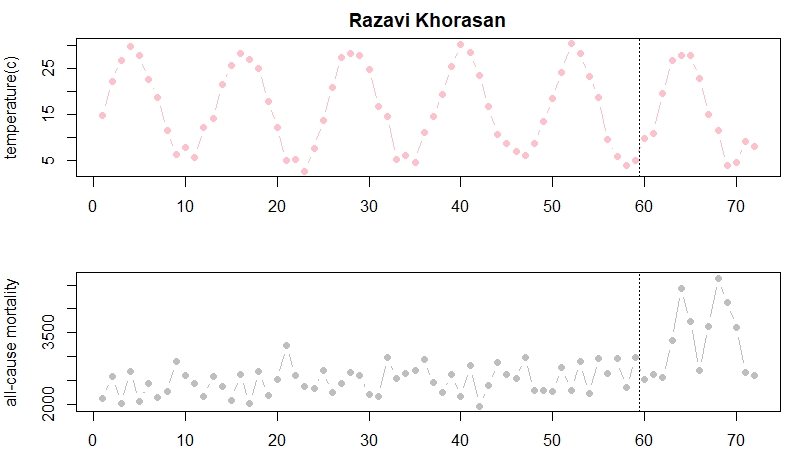


**months**


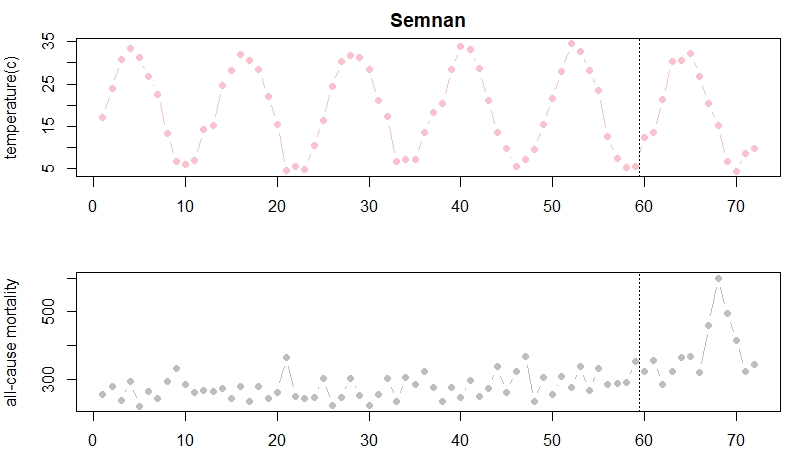


**months**


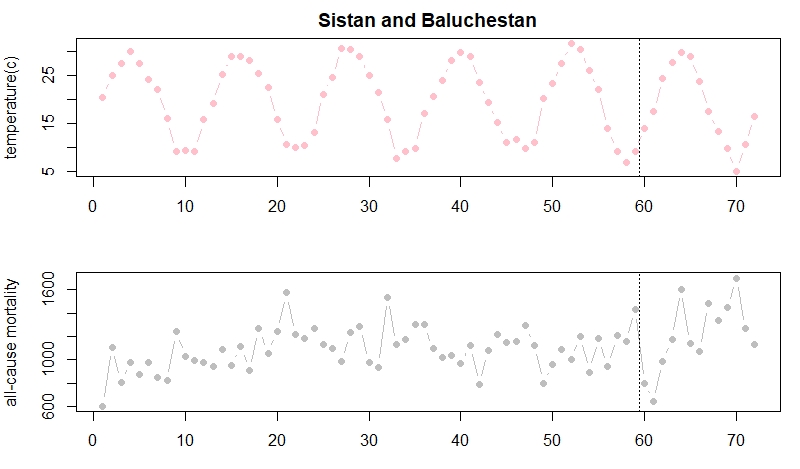


**months**


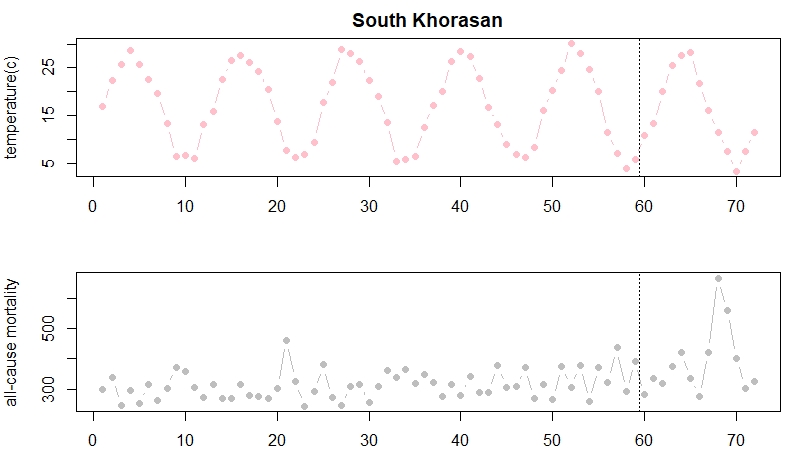


**months**


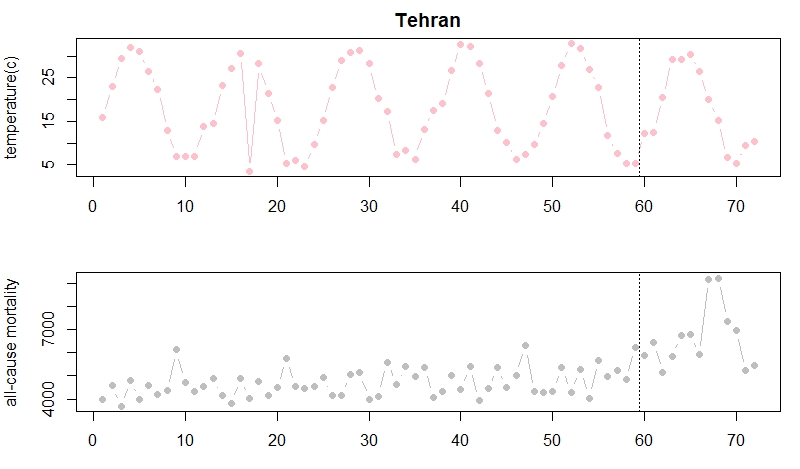


**months**


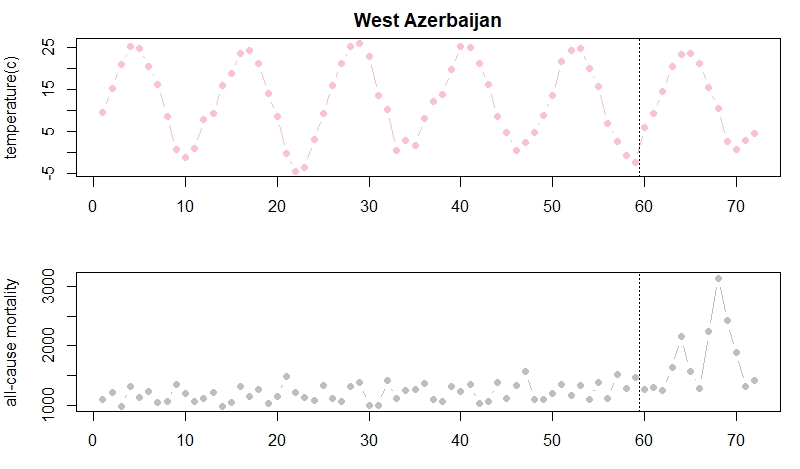


**months**


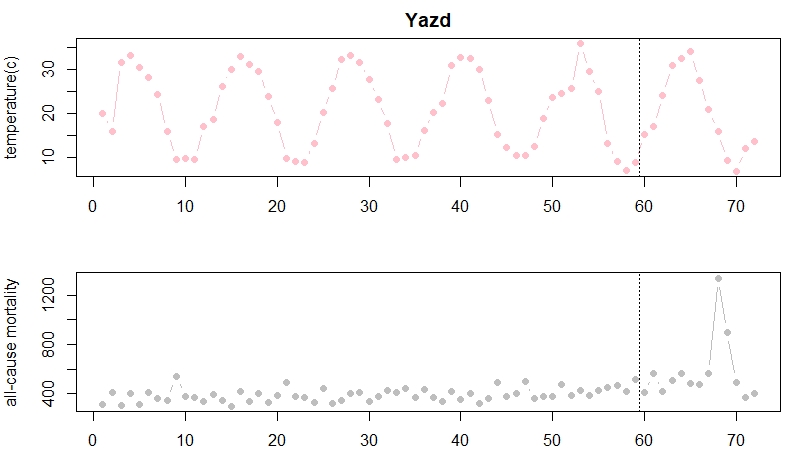


**months**


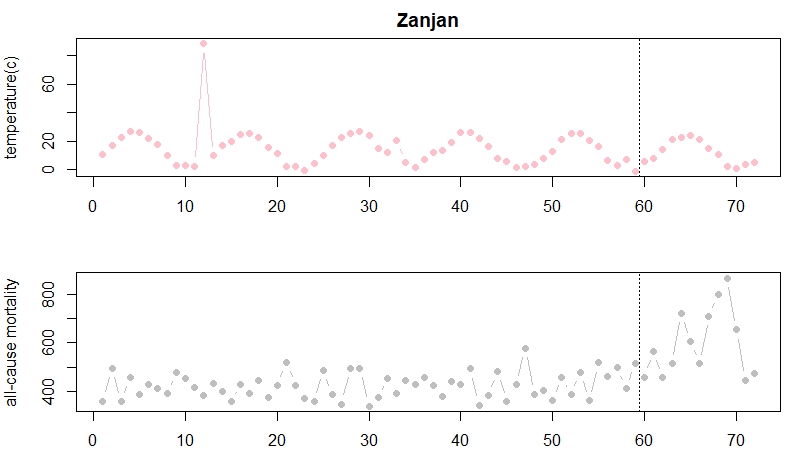


**months**

**Section 3: Sensitivity analysis of potential confounding effect of temperature change on relationship between COVID-19 outbreak and excess all-cause mortlity**

| **Province** | **Excess mortality without controlling for temperature** | **Excess mortality with controlling for temperature** |
| --- | --- | --- |
| Alborz | 4617 | 4603 |
| Ardabil | 2404 | 2415 |
| Bushehr | 1016 | 1023 |
| Chaharmahal and Bakhtiari | 2377 | 2377 |
| East Azerbaijan | 8425 | 8432 |
| Fars | 6778 | 6767 |
| Gilan | 4372 | 7372 |
| Golestan | 3115 | 3120 |
| Hamadan | 2674 | 2669 |
| Hormozgan | 1350 | 1351 |
| Ilam | 938 | 938 |
| Isfahan | 8300 | 8300 |
| Kerman | 3197 | 3201 |
| Kermanshah | 2987 | 2985 |
| Khuzestan | 5824 | 5799 |
| Kohgiluyeh and Boyer-Ahmad | 565 | 565 |
| Kurdistan | 3696 | 3710 |
| Lorestan | 2363 | 2361 |
| Markazi | 2809 | 2809 |
| Mazandaran | 5531 | 5529 |
| North Khorasan | 819 | 810 |
| Qazvin | 2586 | 2602 |
| Qom | 2435 | 2429 |
| Razavi Khorasan | 8995 | 9001 |
| Semnan | 1115 | 1109 |
| Sistan and Baluchestan | 722 | 721 |
| South Khorasan | 665 | 663 |
| Tehran | 20791 | 20813 |
| West Azerbaijan | 6058 | 6058 |
| Yazd | 1930 | 1926 |
| Zanjan | 2067 | 2070 |
