# Supplementary material for: The COVID-19 pandemic’s true death toll in Iran after two years: an interrupted time series analysis of weekly all-cause mortality data
Source: BMC Public Health. 2023 Mar 7;23:442. doi: 10.1186/s12889-023-15336-0 (PMC9990579; doi:10.1186/s12889-023-15336-0)
Supplement: Supplementary file 2 — Supplementary Material 2: The COVID-19 Pandemic?s True Death Toll in Iran after Two Years: An Interrupted Time Series Analysis of Weekly All-Cause Mortality Data [file 12889_2023_15336_MOESM2_ESM.docx]

**Supplementary File**

**The COVID-19 Pandemic’s True Death Toll in Iran after Two Years: An Interrupted Time Series Analysis of Weekly All-Cause Mortality Data**

**Section 1: seasional pattern of all-caused mortality number during the observation period in Iran.**


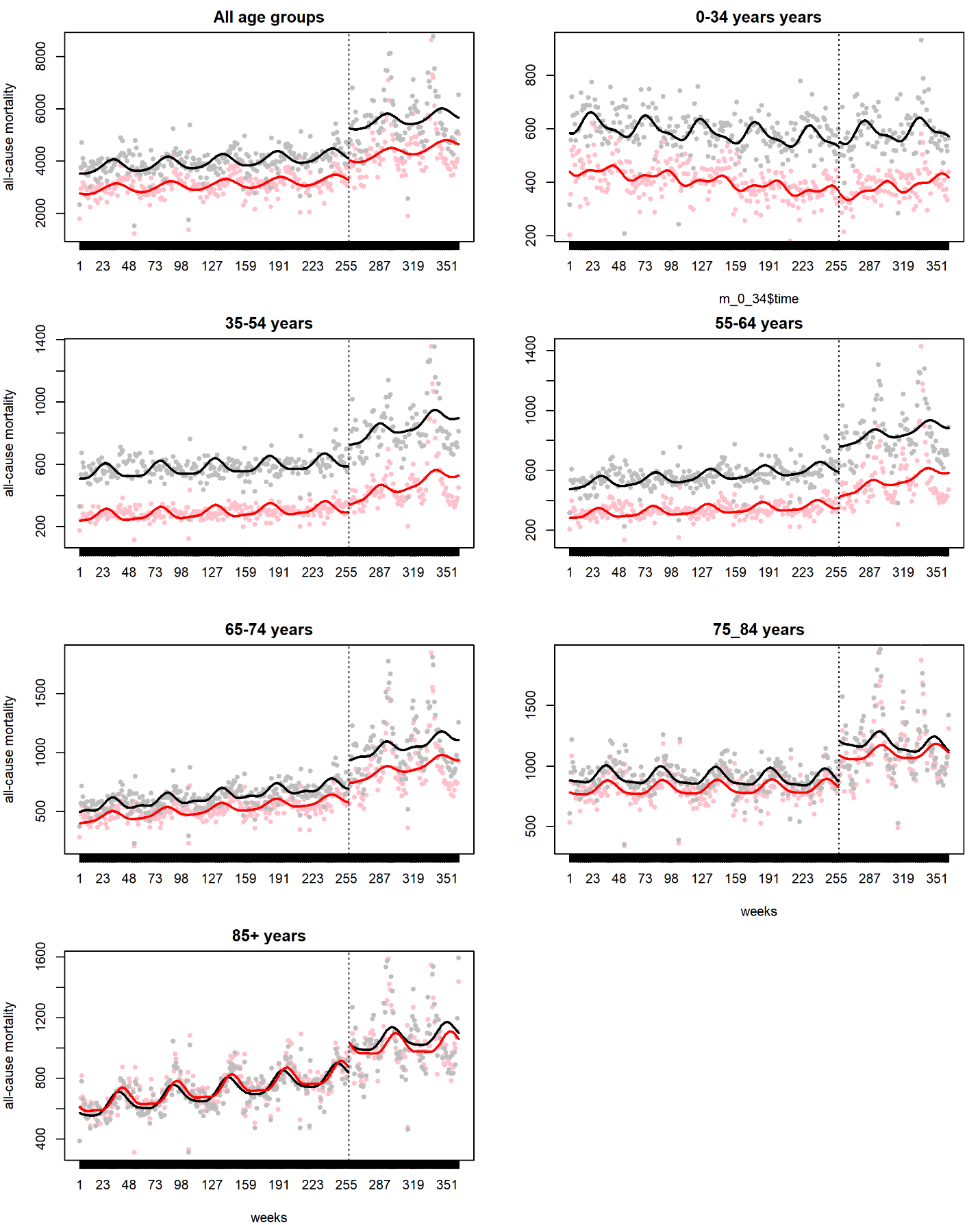


**Section 2: visual inspection of potential relationship between changes in monthly tempreture and monthly all-cause mortality**


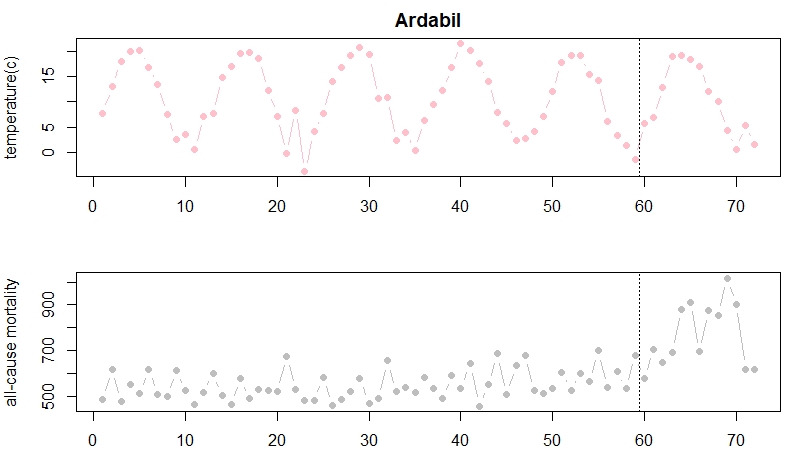


**months**


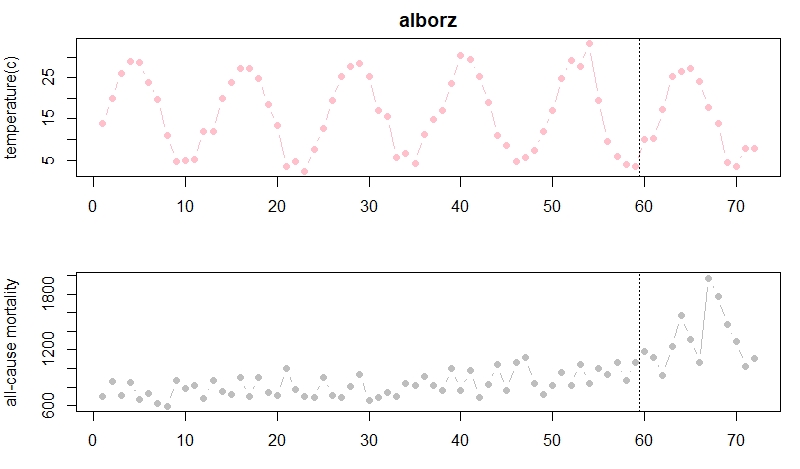


**months**


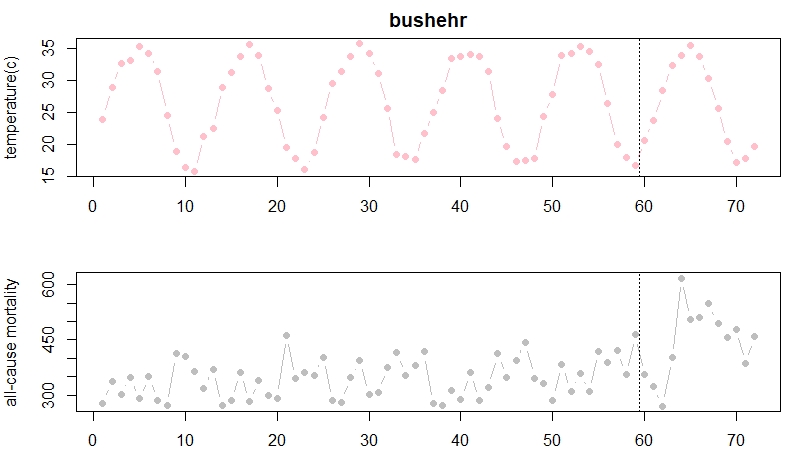


**months**


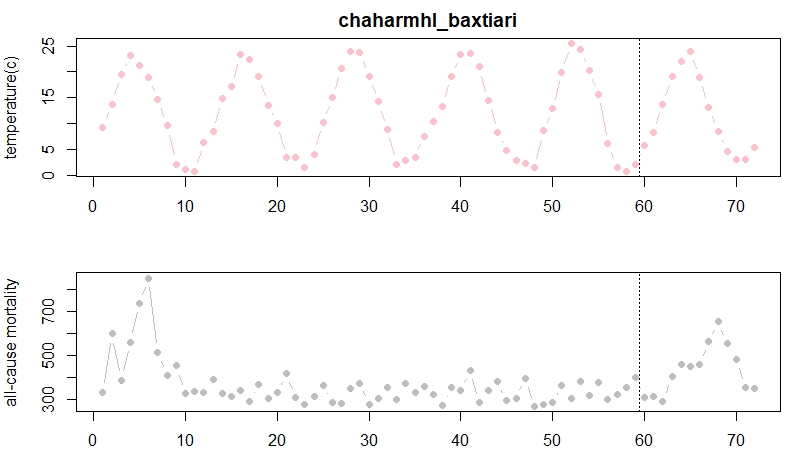


**months**


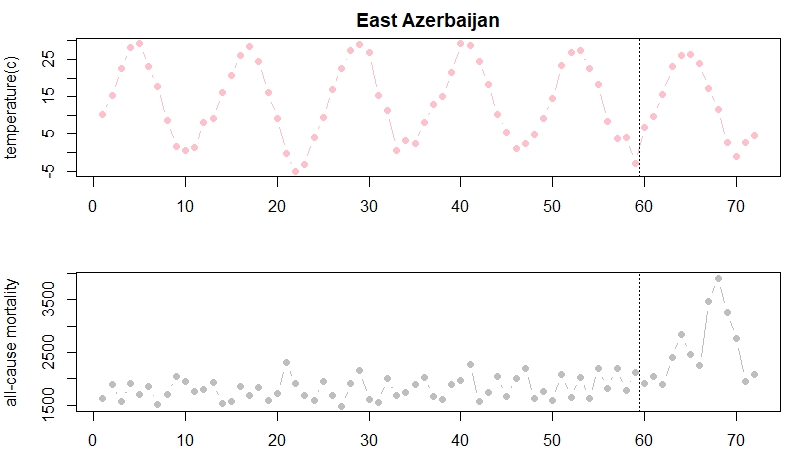


**months**


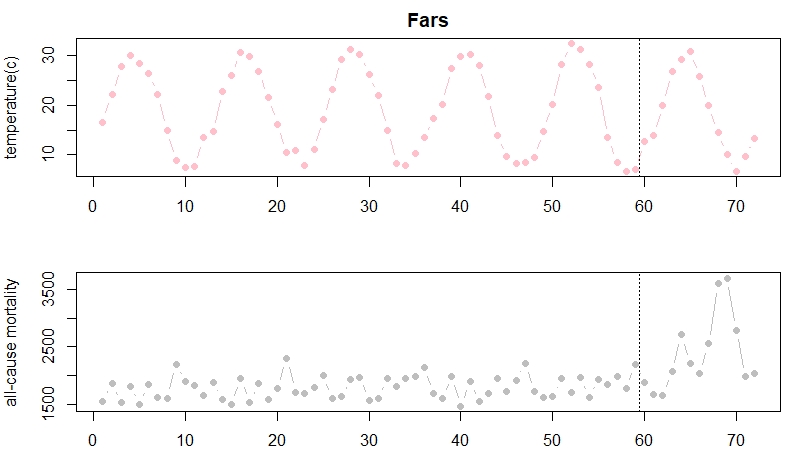


**months**


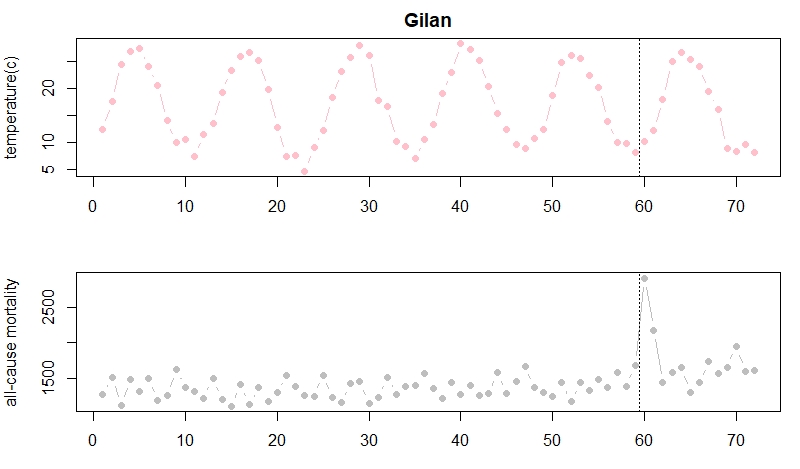


**months**


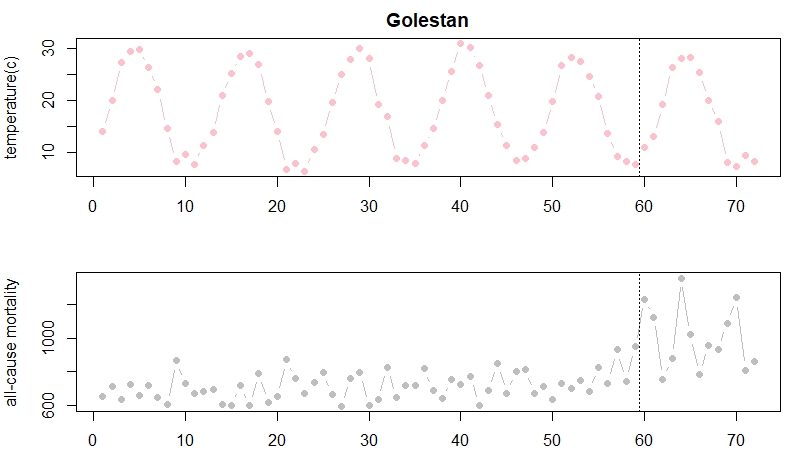


**months**


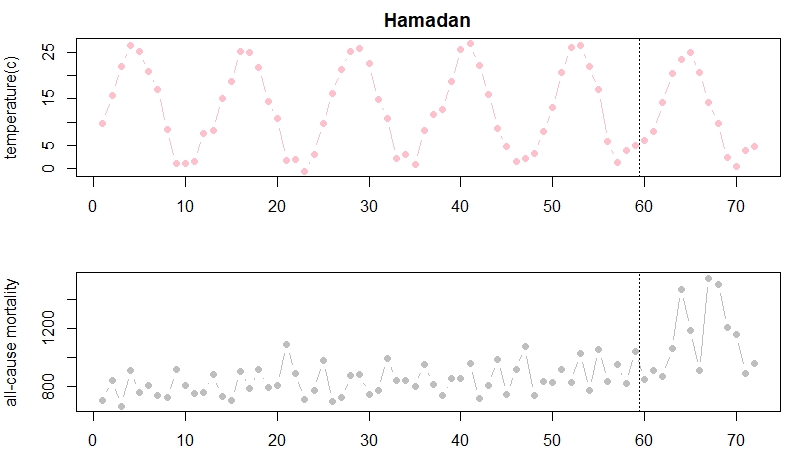


**months**


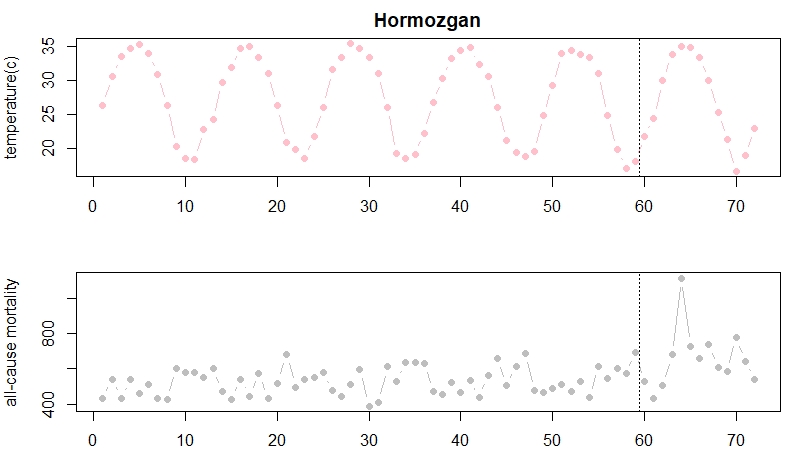


**months**


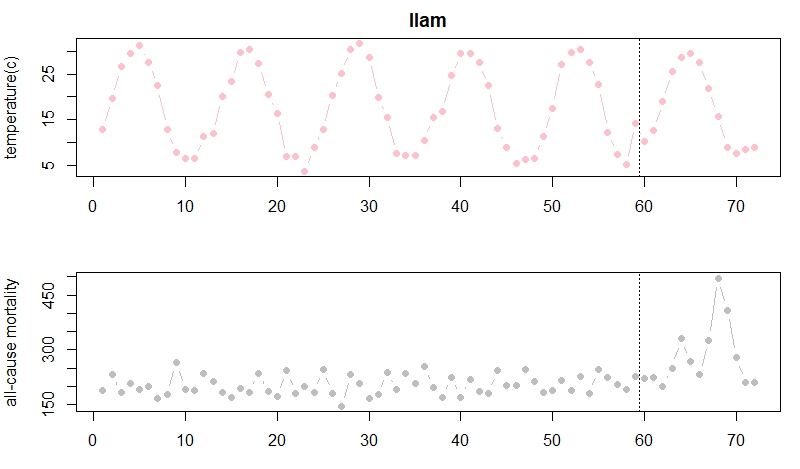


**months**


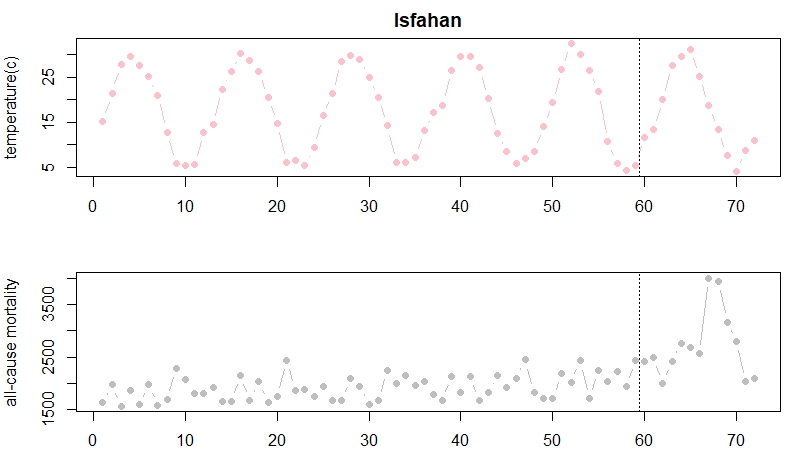


**months**


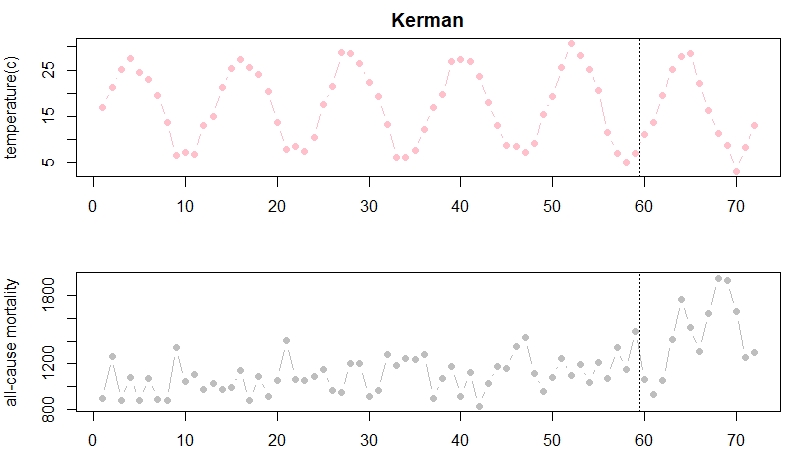


**months**


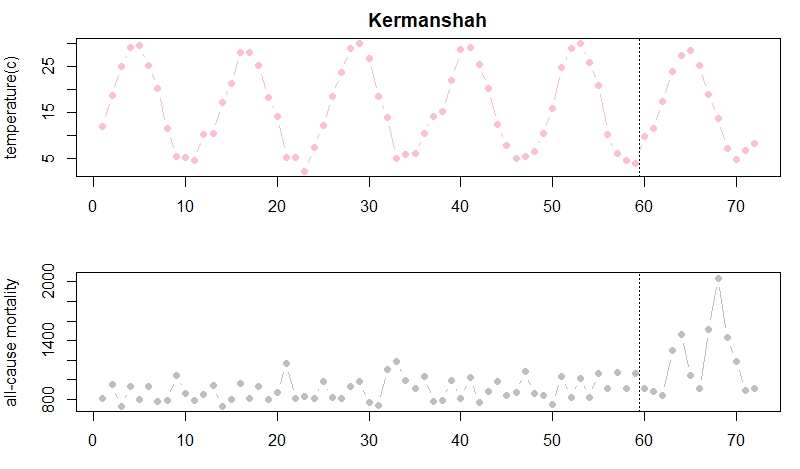


**months**


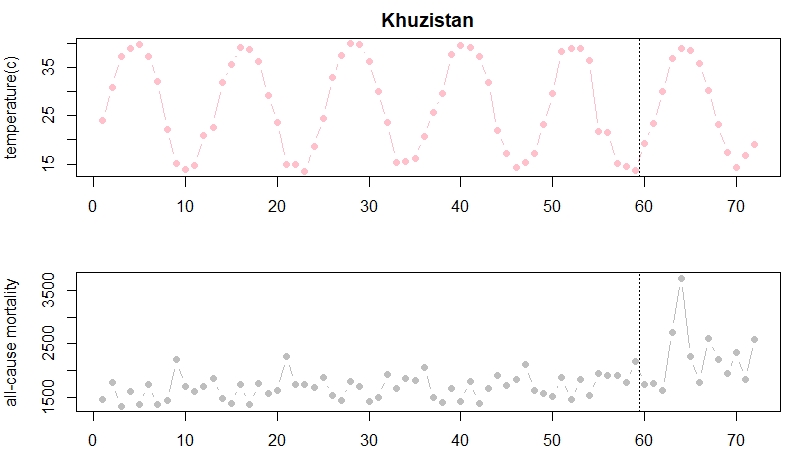


**months**


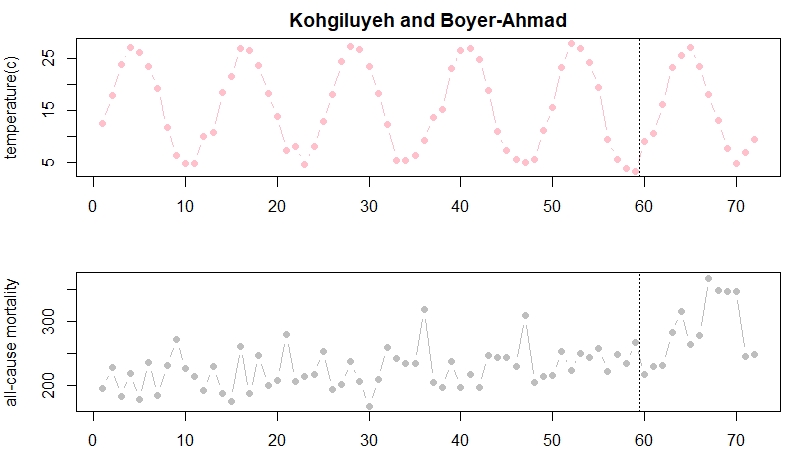


**months**


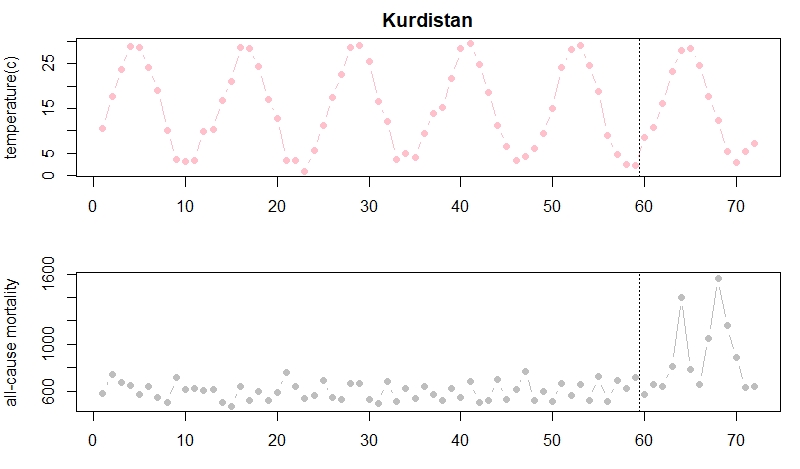


**months**


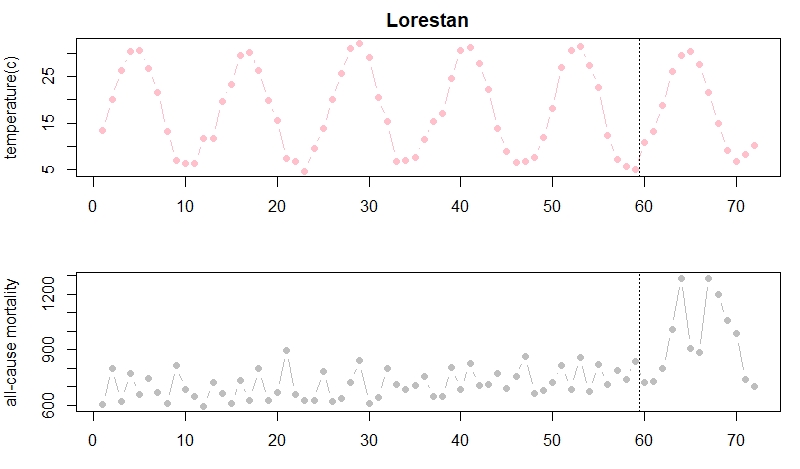


**months**


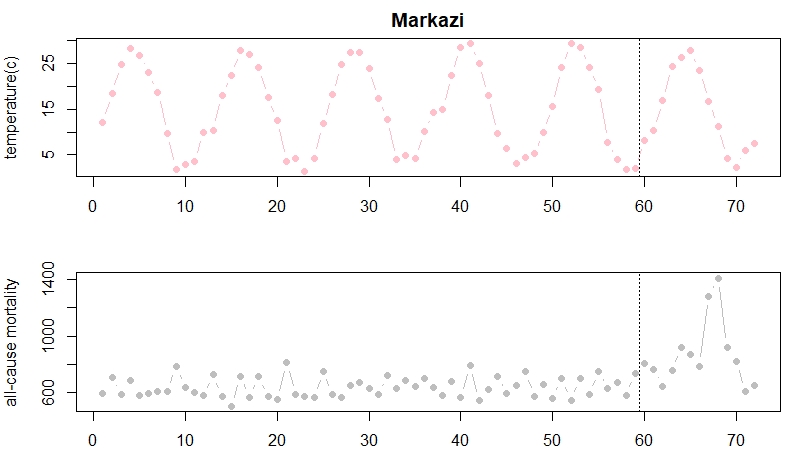


**months**


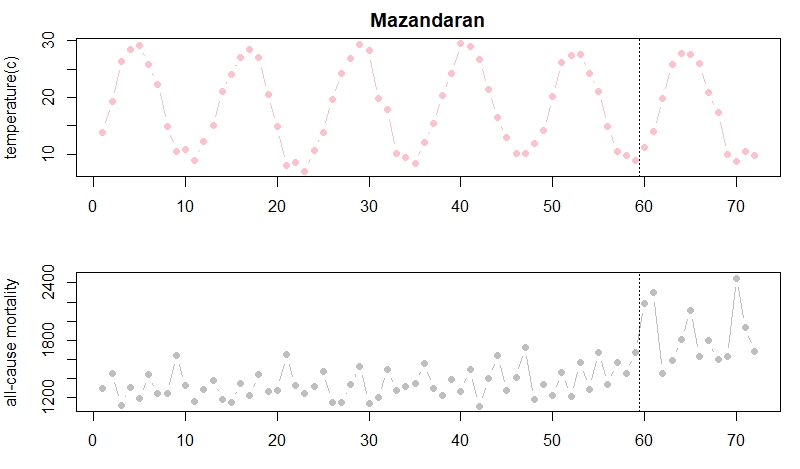


**months**


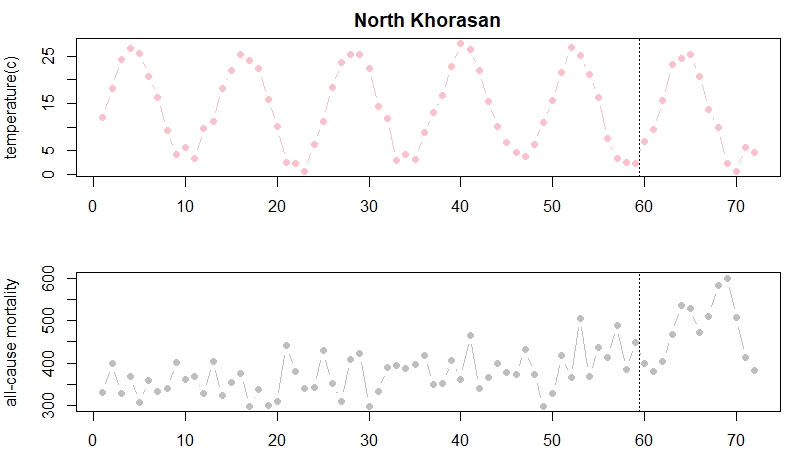


**months**


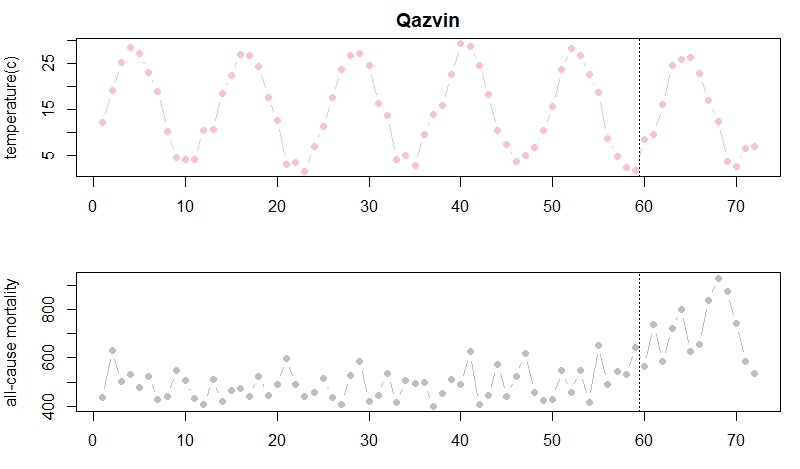


**months**


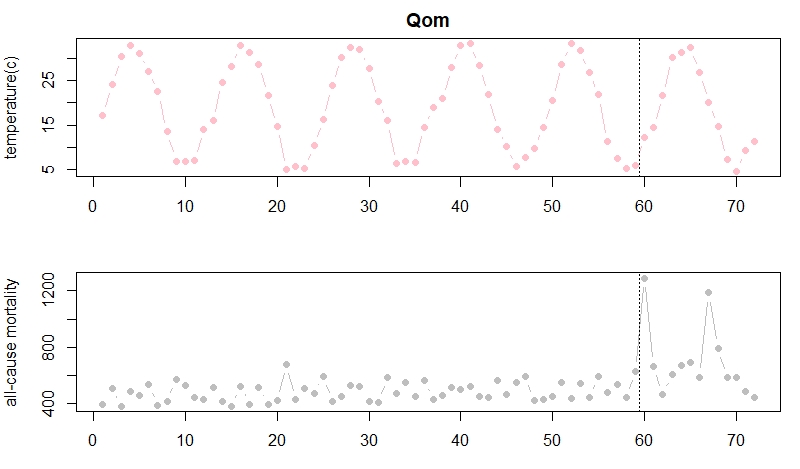


**months**


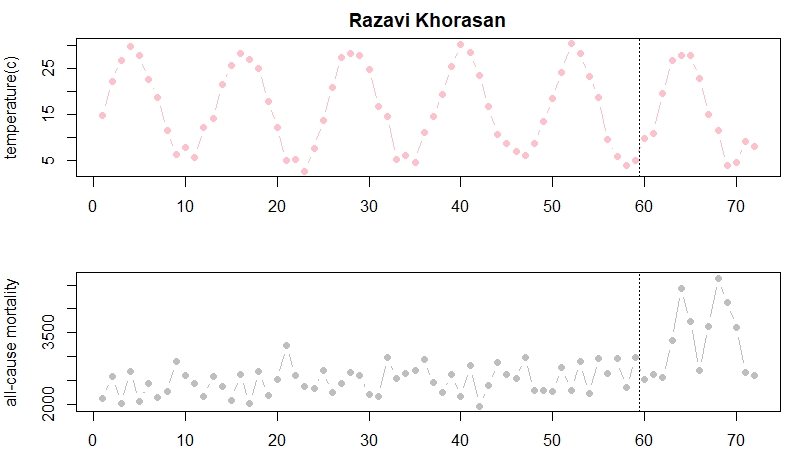


**months**


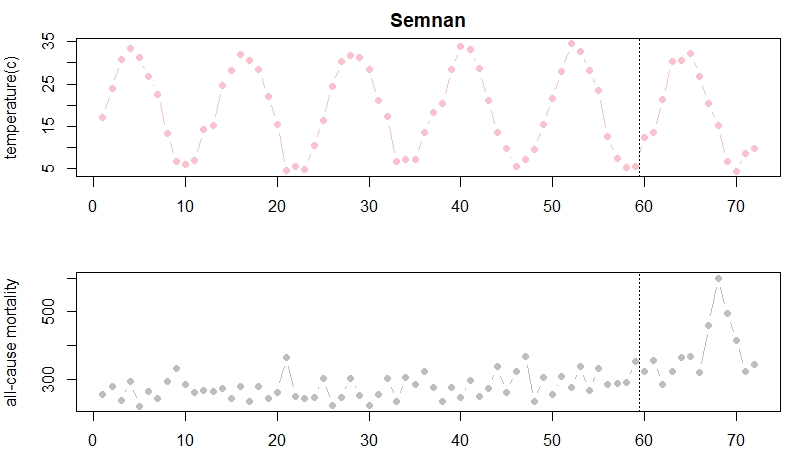


**months**


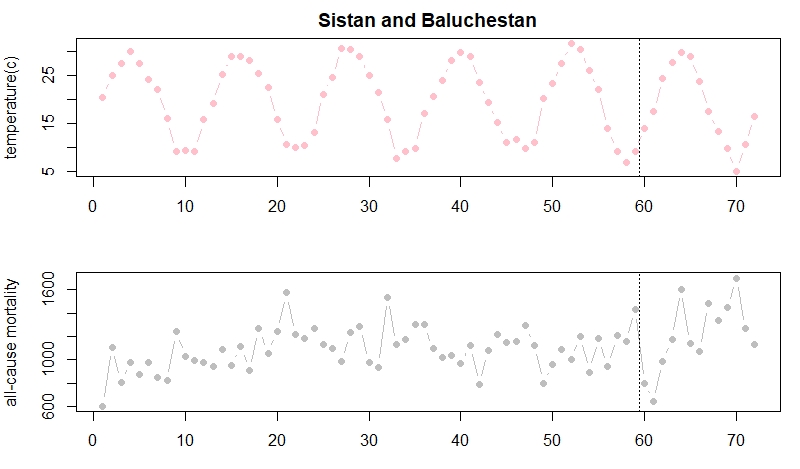


**months**


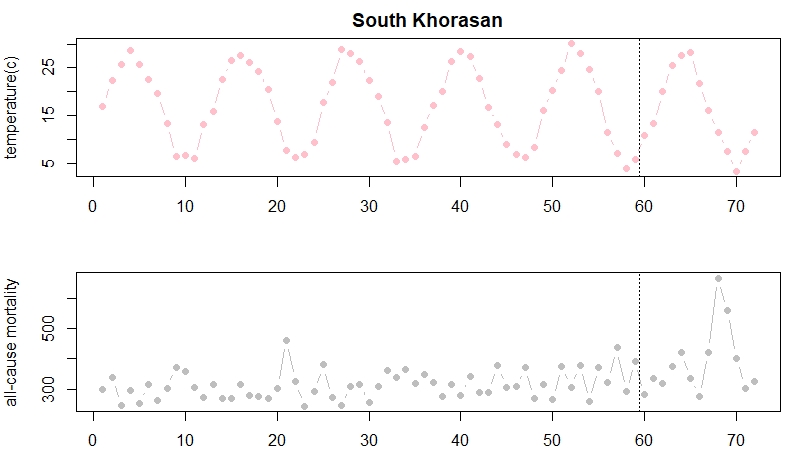


**months**


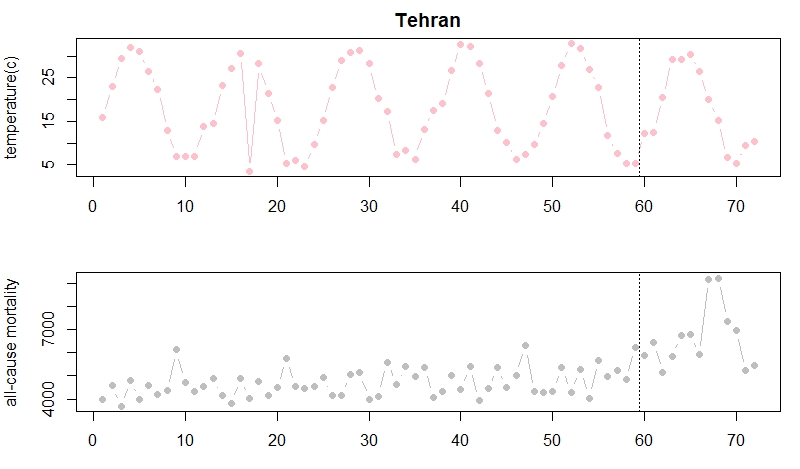


**months**


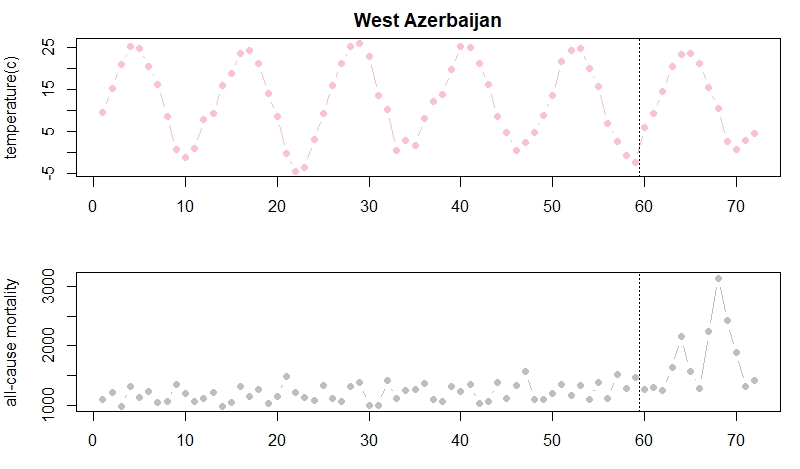


**months**


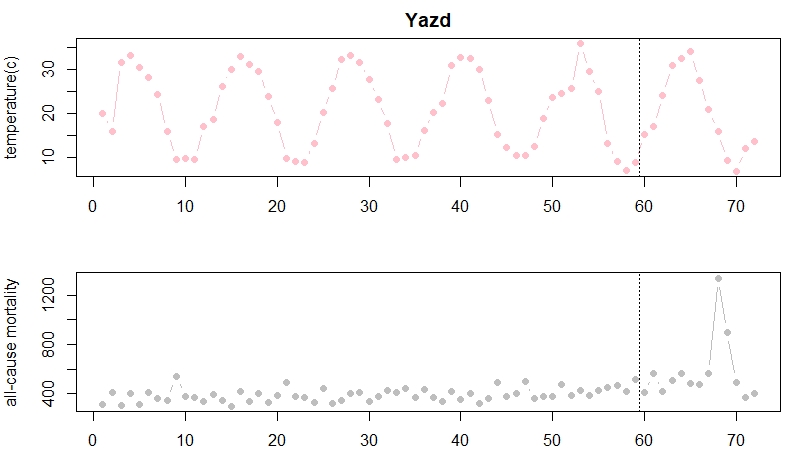


**months**


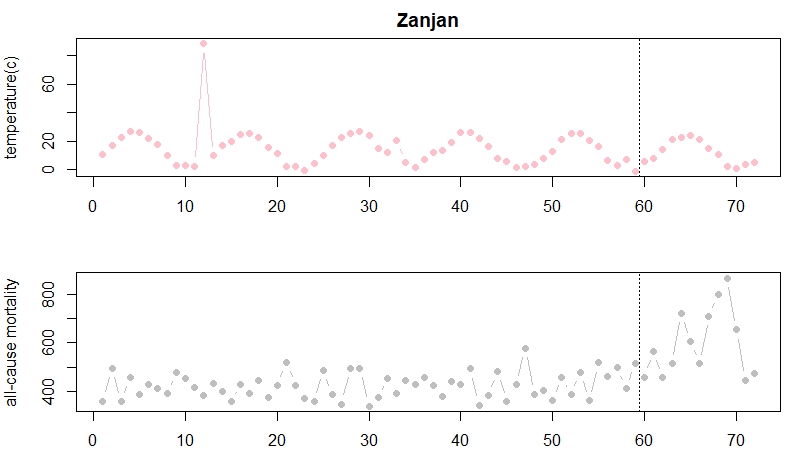


**months**

**Section 3: Sensitivity analysis of potential confounding effect of temperature change on relationship between COVID-19 outbreak and excess all-cause mortlity**

| **Province** | **Excess mortality without controlling for temperature** | **Excess mortality with controlling for temperature** |
| --- | --- | --- |
| Alborz | 4617 | 4603 |
| Ardabil | 2404 | 2415 |
| Bushehr | 1016 | 1023 |
| Chaharmahal and Bakhtiari | 2377 | 2377 |
| East Azerbaijan | 8425 | 8432 |
| Fars | 6778 | 6767 |
| Gilan | 4372 | 7372 |
| Golestan | 3115 | 3120 |
| Hamadan | 2674 | 2669 |
| Hormozgan | 1350 | 1351 |
| Ilam | 938 | 938 |
| Isfahan | 8300 | 8300 |
| Kerman | 3197 | 3201 |
| Kermanshah | 2987 | 2985 |
| Khuzestan | 5824 | 5799 |
| Kohgiluyeh and Boyer-Ahmad | 565 | 565 |
| Kurdistan | 3696 | 3710 |
| Lorestan | 2363 | 2361 |
| Markazi | 2809 | 2809 |
| Mazandaran | 5531 | 5529 |
| North Khorasan | 819 | 810 |
| Qazvin | 2586 | 2602 |
| Qom | 2435 | 2429 |
| Razavi Khorasan | 8995 | 9001 |
| Semnan | 1115 | 1109 |
| Sistan and Baluchestan | 722 | 721 |
| South Khorasan | 665 | 663 |
| Tehran | 20791 | 20813 |
| West Azerbaijan | 6058 | 6058 |
| Yazd | 1930 | 1926 |
| Zanjan | 2067 | 2070 |

**Section 4: Sensitivity analysis of potential confounding effect of applied regression models and excess all-cause mortlity**

| Age group | Sex | Observed post-covid mortality | Total excess mortality (GLS) | Total excess mortality (GLM with Poisson distribution) |
| --- | --- | --- | --- | --- |
| All ages | **Both** | **1050157** | **240390** | **238926** |
|  | Male | 588988 | 134214 | 135025 |
|  | Female | 461169 | 106300 | 103901 |
| 0-34 yr | Male | 61755 | 4426 | 4329 |
|  | Female | 40055 | 4422 | 4332 |
| 35-54 yr | Male | 89010 | 22438 | 21980 |
|  | Female | 49219 | 14132 | 14009 |
| 55-64 yr | Male | 89863 | 22331 | 22401 |
|  | Female | 56471 | 16580 | 16600 |
| 65-74 yr | Male | 111663 | 30771 | 30890 |
|  | Female | 92370 | 24953 | 25029 |
| 75-84 yr | Male | 124987 | 32316 | 32270 |
|  | Female | 116418 | 29465 | 30163 |
| 85+ yr | Male | 111710 | 19580 | 19589 |
|  | Female | 106636 | 14135 | 14200 |
